# Safety and efficacy of CD33-targeted CAR-NK cell therapy for relapsed/refractory AML: preclinical evaluation and phase I trial

**DOI:** 10.1186/s40164-024-00592-6

**Published:** 2025-01-02

**Authors:** Ruihao Huang, Xiaoqi Wang, Hongju Yan, Xu Tan, Yingying Ma, Maihong Wang, Xiao Han, Jia Liu, Li Gao, Lei Gao, Guangjun Jing, Cheng Zhang, Qin Wen, Xi Zhang

**Affiliations:** 1 Medical Center of Hematology, Xinqiao Hospital of Army Medical University, Chongqing, 400037 China; 2 Chongqing Key Laboratory of Hematology and Microenvironment, Chongqing, 400037 China; 3 State Key Laboratory of Trauma and Chemical Poisoning, Army Medical University, Chongqing, 400037 China; 4 iCareab Biotechnology Co., Ltd, Shanghai, China; 5 Jinfeng Laboratory, Chongqing, 401329 China

**Keywords:** CAR-NK, R/R AML, CD33

## Abstract

**Background:**

Due to the lack of effective treatment options, the prognosis of patients with relapsed/refractory acute myeloid leukemia (R/R AML) remains poor. Although chimeric antigen receptor (CAR)-T-cell therapy has shown promising effects in acute lymphoblastic leukemia (ALL) and lymphoma, its application in R/R AML is limited by “off-target” effects, which lead to severe bone marrow suppression and limit its clinical application. CAR-natural killer (NK) cells not only exhibit antitumor effects but also demonstrate increased safety and universality. We have developed a new CAR construct that targets CD33 and modified NK cells, specifically eliminating AML cells while reducing severe side effects on stem cells.

**Methods:**

The CD33-targeting domain was selected by CAR-T cells, and this optimized CAR construct was subsequently transduced into umbilical cord-derived NK cells via a retroviral vector. Preclinical efficacy and safety studies were conducted both in vitro and in vivo. Ten eligible patients with R/R AML aged 18–65 years who received one or more infusions of anti-CD33 CAR-NK cells following the preconditioning regimen were enrolled. We assessed the response rates and treatment-related side effects post-infusion, while also documenting the long-term efficacy of the therapy.

**Results:**

The CD33 sequence was selected on the basis of its antitumor efficacy and safety in CAR-T-cell studies conducted both in vitro and in vivo. CD33 CAR-NK cells demonstrated efficacy comparable to that of CD33 CAR-T cells but showed limited toxicity to hematopoietic stem cells (HSCs). Ten patients, with a median of five prior lines of treatment, completed the efficacy evaluation (range, 3–8). No grade 3–4 adverse events were observed, except bone marrow suppression, which was relieved within one month. No cases of immune effector cell–associated neurotoxicity syndrome (ICANS) or graft-versus-host disease (GVHD) were reported following CAR-NK cell infusion. Only one patient experienced grade 2 cytokine release syndrome (CRS) and presented with persistent fever. By day 28, six of ten patients had achieved minimal residual disease (MRD)-negative complete remission.

**Conclusions:**

Our preclinical and clinical data demonstrated the primary efficacy and safety of CD33 CAR-NK cells for patients with R/R AML. Expanded samples and longer follow-up periods are needed to provide further efficacy data.

**Trial registration:**

NCT05008575 (https://clinicaltrials.gov/study/NCT05008575).

**Supplementary Information:**

The online version contains supplementary material available at https://doi.org/10.1186/s40164-024-00592-6.

## Introduction

Acute myeloid leukemia (AML) is the most common form of acute leukemia in adults, with a dismal 5-year survival rate of only 24%. This prognosis is even worse in refractory or relapsed (R/R) patients, where the 5-year survival rate falls below 10% [1]. Given these bleak statistics, new therapeutic strategies are urgently needed. CD19 chimeric antigen receptor (CAR)-T-cell therapy has significantly improved outcomes in B-cell malignancies [1–4], and several CD19 CAR-T-cell therapies have been approved by drug administration facilities worldwide [1]. However, clinical trials related to CAR-T-cell therapies for R/R AML have shown modest results [2]. One potential challenge in applying CAR-T-cell therapy to AML is the limited availability of suitable targets for malignant myeloid cells. The targets for AML treatment can be divided into tumor-associated antigen (TAA) and tumor-specific antigen (TSA) targets [1]. TSAs typically arise from the abnormal expression of specific proteins or antigens, such as the Lewis Y antigen. However, TSA-targeted CAR-T-cell therapy in AML has shown limited antitumor efficacy because of the incomplete expression of TSAs across all AML cells, leading to a high incidence of target-negative recurrent disease. In TAA-targeted CAR-T-cell therapy, the expression of TAAs on hematopoietic stem cells (HSCs), such as CD33, can lead to on-target, off-tumor side effects, often manifesting as bone marrow suppression [1, 3]; these effects are more severe than those of B-cell line or plasma cell deficiency in lymphoid malignant cells. For mitigation of these life-threatening side effects, AML CAR-T-cell therapy has been designed as a bridge to hematopoietic stem cell transplantation (HSCT), allowing the elimination of CAR-T cells while preserving hematopoietic function. This approach addresses a key concern that has limited the broader application of CAR-T-cell therapy [4]. This approach relies on modifying T cells isolated from the peripheral blood of AML patients, which presents challenges in achieving the comparable response rates observed in B-cell acute lymphoblastic leukemia (B-ALL). The activated CAR-T secreted cytokines like GM-CSF, FLT3L and IL-3 will induce the exhaustion of T cells in AML [5]. Therefore, other immune cells should be explored.

Natural killer (NK) cells play a crucial role in innate immune surveillance and can be utilized without the need for human leukocyte antigen (HLA) matching [6]. Haploidentical NK cells have been clinically employed in the treatment of myelodysplastic syndrome and AML, demonstrating a favorable safety profile but limited antitumor efficacy. In contrast, CD19 CAR-NK cells have shown promising antitumor activity in indolent lymphomas [7]. Cell lines like NK92 derived CAR-NK cells have manifested satisfying safety but inefficient efficacy [8].Additionally, CAR-NK cell therapy has shown efficacy in several cancers and is further optimized by the inclusion of cytokines such as IL-15 [9–12]. Therefore, we designed a CAR structure that targets CD33 with soluble IL-15 and used NK cells to replace T cells to prevent prolonged bone marrow depression and provide universal immune cell therapy.

To increase the efficacy of NK cells derived from umbilical cord blood, we developed a novel anti-CD33 CAR epitope that incorporates a 4-1BB costimulatory domain and a soluble IL-15 sequence; this construct was transduced via a lentiviral vector. In an animal model, CD33 CAR-NK cells demonstrated increased antileukemia efficacy, comparable to that of CD33 CAR-T-cell therapy. We designed a phase I clinical trial to evaluate the safety and preliminary efficacy of CD33 CAR-NK cells in AML.

## Materials and methods

### Cell isolation and culture

T cells were derived from the peripheral blood mononuclear cells (PBMCs) of healthy donors with informed consent. NK cells were isolated from cord blood mononuclear cells (CBMCs) and then cocultured with genetically engineered K562 feeder cells and IL-2 to promote directed expansion. On day 4, the cells were transduced with lentiviral vectors encoding an anti-CD33 CAR, the 4-1BB-CD3ζ signaling intracellular domain, a P2A self-cleaving peptide, and mature IL-15. The NK cells were continuously expanded until day 15, at which point the CAR-NK cells were cryopreserved via CS10 reagent (Supplement 1).

### In vivo animal model

Animal studies were performed under protocols approved by the Institutional Animal Care and Use Committees (IACUCs) of the Xinqiao Hospital of Army Medical University. Male NSG (NOD.Cg-PrkdcscidIL2rgtm1Wjl/SzJ) mice, aged 6–8 weeks, were purchased from The Jackson Laboratory (No. 005557) and used for all xenograft experiments. K562 and Molm13 AML cells (provided by Cobioer Company, Nanjing) were stably transfected with a retroviral vector expressing GFP and luciferase. These cells were cultured in IMDM and RPMI-1640 medium (Gibco) supplemented with 10% FBS. For the Molm13-luc AML models, 3.3 × 10^5^ Molm13-luc cells in 200 µL of PBS were injected into the tail vein. Tumor size was measured with bioluminescence for randomization before CAR-T-cell transfer and then twice per week to monitor tumor growth. B6129SF1/J mice received 1.5 × 10^6^ CAR-T or 1 × 10^7^ CAR-NK cells via the tail vein. On day 21, the mice without detectable tumors (~ 75% of the cohort) and the age-matched naive control mice received 3.3 × 10^5^ Molm13-luc cells via the tail vein and 1.5 × 10^6^ T cells.

### CD33 sequence selection

CD33 sequences were selected from a CAR-T-cell model on the basis of the antitumor efficacy of CD33-overexpressing Molm13-luc cells and the results of a hematopoietic toxicity assay.

### CD33 CAR-NK cell generation

The process for generation of CAR-NK cells was as follows. Frozen CBMCs were thawed, and NK cells were purified via CD3-negative selection and cultured in serum-free medium containing IL-2. On day 8, the NK cells were transduced with a lentiviral vector carrying the CD33 CAR gene. The cells were expanded for an additional 7 days and harvested on day 15.

### Study design

**Fig. 1:**
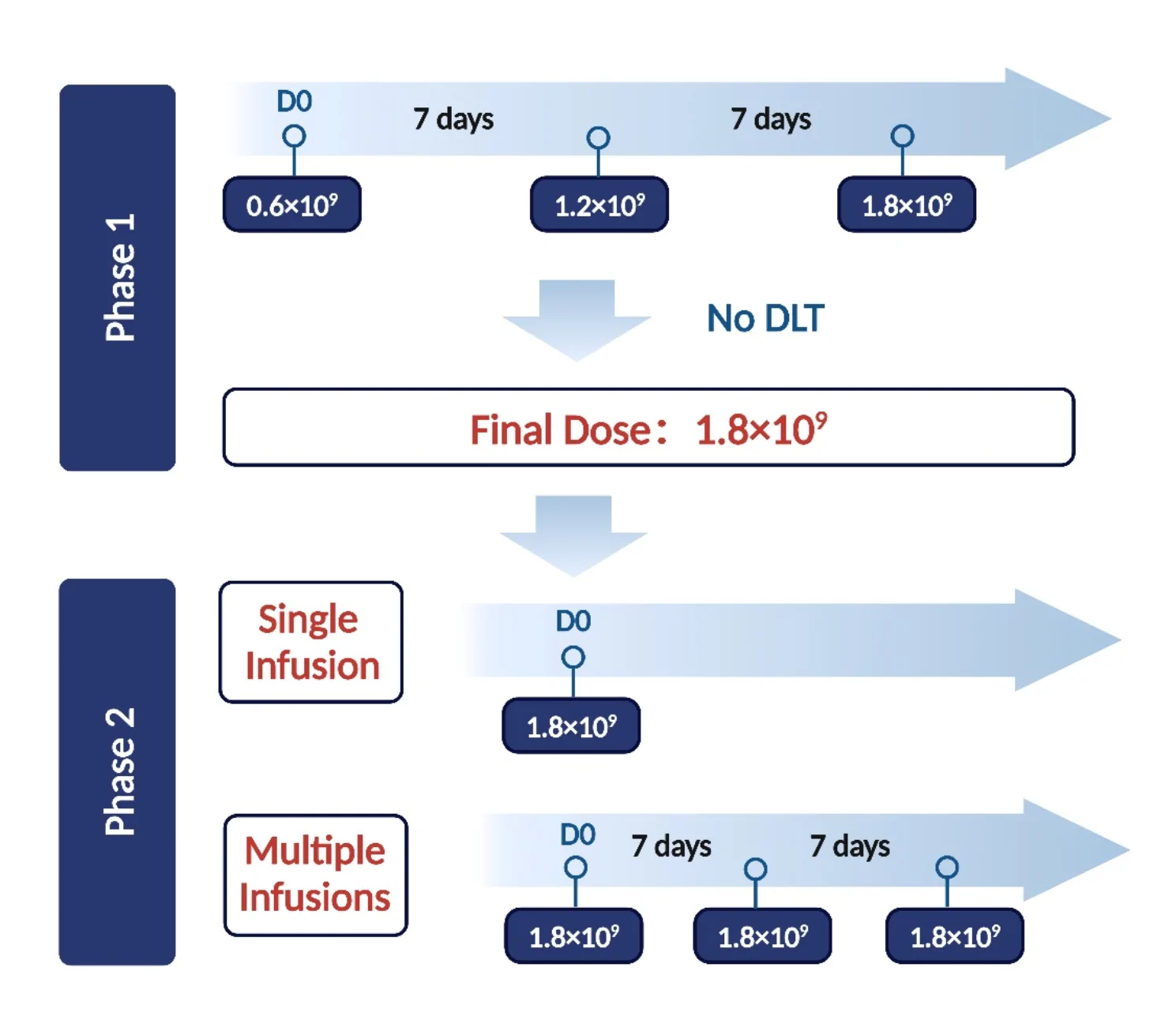
Clinical study schema

Twelve patients were recruited between December 2021 and June 2022 at Xinqiao Hospital of Army Medical University (the clinical trial protocol is provided as separate supplementary information). After 3 days of lymphodepletion preconditioning [fludarabine (30 mg/m^2^) and Cytoxan (300–500 mg/m^2^)], one or more rounds of CAR-NK cells were infused. In dose group one, patients first received 6 × 10^8^ CAR-NK cells, and adverse events (AEs) were carefully recorded. On day 7, the dose was increased to 1.2 × 10^9^ CAR-NK cells for infusion if the patient presented no severe AEs; 14 days after the first infusion, the dose was further increased to 1.8 × 10^9^ CAR-NK cells. Three patients received 3 rounds of CAR-NK cells (6 × 10^8^, 1.2 × 10^9^ and 1.8 × 10^9^ cells) at intervals of 7 days, and any AEs were recorded. In the dose expansion groups, 3 patients received one dose of 1.8 × 10^9^ CD33 CAR-NK cells. Four patients received 3 rounds of 1.8 × 10^9^ CD33 CAR-NK cells at intervals of 7 days (Fig. 1). Clinical responses to therapy were based on the 2003 International Working Group (IWG) response criteria for AML.

The study was approved by the Ethics Committee of Xinqiao Hospital and conducted in accordance with the principles of the Declaration of Helsinki (NCT05008575). Informed consent was obtained from all patients. All the authors confirmed the completeness and accuracy of the reported data and AEs and ensured that the study adhered to the established protocol.

#### qPCR

Cellular genomic DNA (gDNA) was extracted from 1 ml of whole blood using the QIAamp DNA Blood Midi Kit. One hundred nanograms of DNA was amplified using the TB Green Premix ExTaq (Tli RnaseH Plus) Kit with the amplification primer pairs, premixed TB Green Premix Ex TaqII (Tli RnaseH Plus) solution and rOX reference dye following the manufacturer’s recommended protocols. Amplification was detected in real time using the Applied Biosystems 7500 Real-Time PCR System. A primer pair targeting the 3’ LTR region was used to measure the CAR copy number.

NK-WPRE-F2: CACACAA GGCTACTTCCCTGAT.

NK-WPRE-R2: GGCTCAACTGGTACTAGCTTGT.

NK-WPRE-P2: TAGCAGAAC TACACACCAGGGCCAGG.

These primers are used for the CAR-NK (3’ LTR) and are applicable to CD33 CAR-NK projects.

### Statistical analysis

We used the Wilcoxon rank-sum test to test the associations between the response to therapy and the quantity of CAR-NK cells. A *P* value of less than 0.05 was considered to indicate statistical significance.

## Results

### Selection of the optimal single-chain variable fragment (scFv) of anti-CD33 by CAR-T cells

**Fig. 2:**
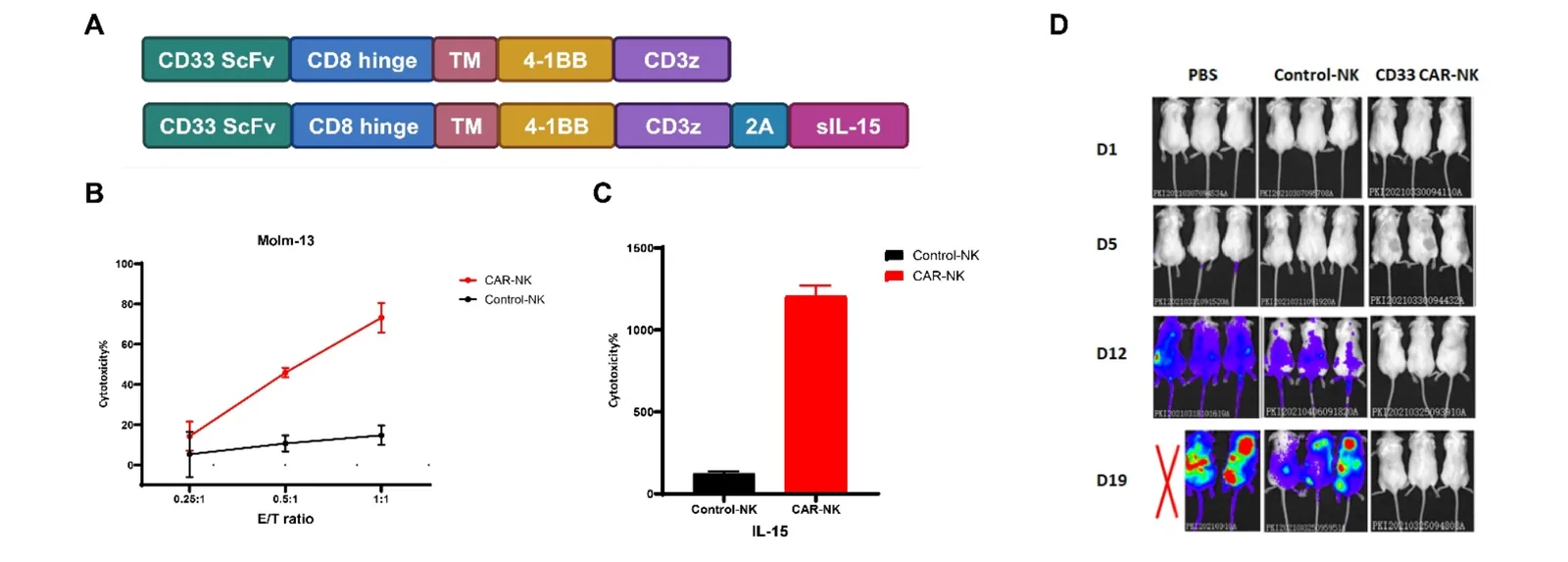
CAR structure and antileukemic efficacy of CAR-NK cells in vitro and in vivo. **A** CAR structure of CAR-T and CAR-NK cells; TM: transmembrane domain. **B** Cytotoxicity assay of CAR-NK cells with Molm-13 cells to show the killing efficacy of CAR-NK cells. **C** Concentrations of soluble IL-15 (E: T 1:1) in the control NK and CD33 CAR-NK cells. **D** Antitumor efficacy of CD33 CAR-NK cells in vivo

Previous work has identified a series of high-affinity antibody‒drug conjugates (Mylotarg) with binding specificity to distinct extracellular domains of CD33. We initially sought to develop a highly functional CAR against CD33 by cloning scFvs derived from the heavy- and light-chain pairs of these antibodies into a retroviral CAR vector with a Flag-tag for easy detection. This vector contained the 4-1BB stimulation domain. We tested scFvs derived from Mylotarg and novel sequences 1 and 2, and isolated T cells from healthy donors were transduced with each CAR (Fig. 2A, Supplement 2). The lysis potential of CAR-T cells was determined with a 20-hour luciferase killing assay with CD33^+^ Molm13 cells. Untransduced T cells were used as a negative control. We found substantial variation in the tumor cell lysis capacity of the CARs, with better performances of 1 and 2 than the control. We then cocultured CAR-T cells with Molm13 cells to assess CD33 CAR-T-cell activation and the proliferative ability. The levels of IL-2, IFN-γ, and TNF-α in the supernatant were measured 24 h after coculture, and CAR-T-cell proliferation was quantified after 7 days. Compared with the control, both the 1 CAR and the 2 CAR showed better efficacy in vitro. Then, we injected Molm13-luc cells into NSG mice to establish an AML model, and the mice were treated intravenously with 1.5 × 10^6^ CAR-T cells on Day 7 after tumor development. Compared with the negative control and the conventional CD33 CAR structure, T cells expressing either the 1 or 2 CAR-binding domains elicited an antitumor response in the AML model (Supplement 3). However, seq-1 showed significantly greater toxicity to HSCs, resulting in a significantly decreased number of burst-forming unit-erythroid, colony-forming unit-granulocyte, colony-forming unit-granulocyte, erythrocyte, monocyte and megakaryocyte cells in the hematopoietic toxicity assay (Supplement 4).

### Umbilical blood cell-derived CAR-NK cell generation

To provide an off-the-shelf and safe immune cell treatment, based on efficacy and safety experiments in an animal model, we transduced sequence 2 into umbilical-derived NK cells to increase antitumor efficacy; a soluble IL-15 and P2A self-cleaving peptides were added to CD33 CAR-NK cells to increase antitumor efficacy in a coculture model (Fig. 2B), and increased levels of IL-15 were observed in vitro (Fig. 2C). In the Molm13 model, tumors were eliminated at 12 days after CAR-NK-mediated treatment (Fig. 2D). In general, CD33 CAR-NK-cell therapy is safe and effective for treating AML in vivo and in vitro.

### Patient characteristics

Between December 2021 and June 2022, 12 patients were screened, 2 of whom were ineligible for NK cell infusion due to fatal infection and disease progression. Ten patients received one or more rounds of CAR-NK cell infusion. The median age of the patients was 42.5 (range, 18–65) years. All ten patients suffered from confirmed R/R AML after a median of 5 (range, 3–8) lines of treatment, and three patients received allogeneic HSCT (allo-HSCT), one patient received Mylotarg, and two patients had secondary AML (1 with myelodysplasia-related changes and 1 with confirmed lung cancer). Six patients had mutations associated with a poor prognosis. The patients’ baseline data are listed in Table 1. The efficacy of the final CAR-NK transduction for the infused product was 49.0% (range, 22.7–66.5%).

**Table 1:** Patient characteristics

No.	Age	Gender	Diagnosis	Lines of treatments	Failure of HSCT or CD33 mAB	Mutation
1	18	F	AML-M2	6	allo-HSCT	/
2	43	F	AML-MRC	3		RUNX1
3	42	F	AML-M2	3		DNMT3A, FLT3-ITD
4	46	F	AML-M2	5		/
5	65	F	AML-M5	8	Mylotarg	AML1-ETO/ABL+
6	26	M	AML-M2	5		FLT3-ITD
7	29	M	AML-M2	7	allo-HSCT	Complex karyotype: 46XY, del(5),(q31),del(15)(q15)
8	35	F	AML-M2	5	allo-HSCT	/
9	52	M	sAML-M1	3		/
10	53	M	AML-MRC	3		WT1, RUNX1, complex karyotype

Footnotes: AML: acute myeloid leukemia; AML-MRC: acute myeloid leukemia with myelodysplasia-related changes; allo-HSCT: allogeneic hematopoietic stem cell transplantation

### Safety

After the infusion of CD33 CAR-NK cells, no immune effector cell–associated neurotoxicity syndrome (ICANS) or graft-versus-host disease (GVHD) was observed. Seven (70%) patients developed fever within two days after the first round of CAR-NK cell infusion; in 6 of the 7 patients, the fever was alleviated after symptomatic treatment. After the infusion of the second dose, a single patient developed recurrent fever, which was alleviated after one dose of 5 mg dexamethasone administered intravenously on the sixth day. No AEs above grade 3 were observed beyond hematological toxicity. None of the patients needed to be transferred to the intensive care unit (ICU). Severe bone marrow depression was self-limiting in the responding patients after a median of 27 days (range, 24–32 days) of supportive treatment (Table 2).

**Table 2:** Adverse events

Adverse Event	Grade 1 or 2	Grade 3	Grade 4
	No. of Patients		
CRS	1	0	0
GVHD	0	0	0
ICANS	0	0	0
Cardiovascular event			
Chest pain	2	0	0
Tachycardia	2	0	0
Infection (bacterial)	2	1	0
Constitutional event			
Fatigue	4	0	0
Insomnia	3	0	0
Gastrointestinal			
Nausea	2	0	0
Diarrhea	1	0	0
Stomachache	1	1	0
Laboratory values			
Hyperaminotransferasemia	2	1	0
Elevated AST	1	2	0
Hyperglycemia	0	1	0
Hyperkalemia	0	1	0
**Hematological Event**	**Grade 1 or 2**	**Grade 3**	**Grade 4**
	**No. of Patients**		
Neutropenia	5	1	4
Lymphopenia	0	3	7
Thrombocytopenia	3	1	5
Anemia	3	5	0

Footnotes: CRS: cytokine release syndrome; GVHD: graft-versus-host disease; ICANS: immune effector cell-associated neurotoxicity syndrome; AST: aspartate aminotransferase

### Treatment response

**Fig. 3:**
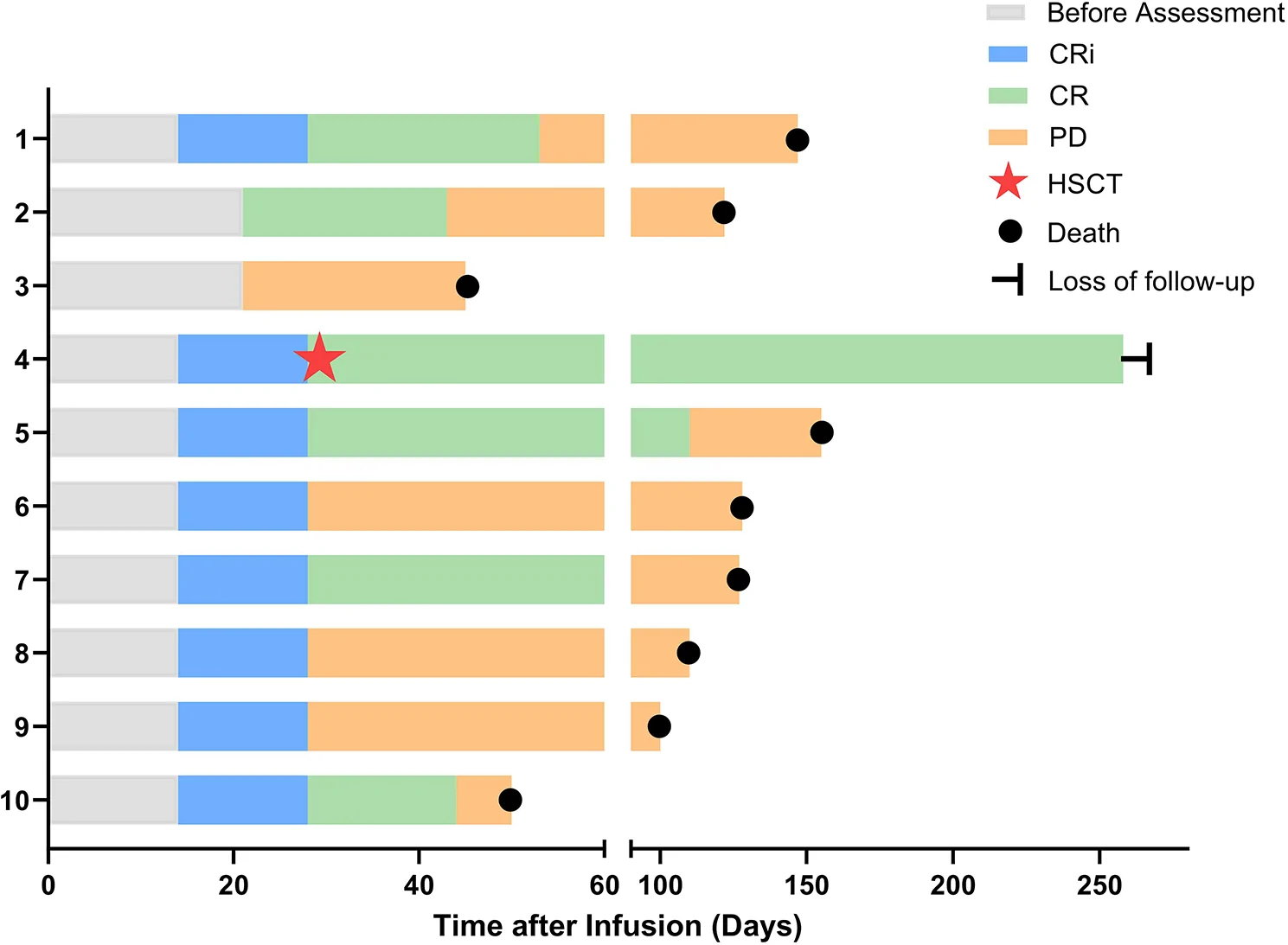
Data for survival after CD33 CAR-NK cell treatment for patients with R/R AML enrolled in the clinical study

The median length of follow-up was 125 days (range, 45–258 days), and six of ten (60%) patients achieved minimal residual disease–negative complete response (MRD-CR), as indicated by imaging (imaging complete response, or iCR), at the day 28 assessment [13]. No significant difference was observed between the three dose groups (*P* = 0.93). Thus, most responses appeared within one month. Only one patient who bridged to allo-HSCT achieved long-term remission (258 days); the other patients had recurrent fatal disease that did not respond to further treatment. The median progression-free survival (PFS) was 71.5 days (range, 44–258 days), and the overall survival (OS) was 137 days (range, 50–258 days) for patients with complete remission (Fig. 3).

### CAR-NK dynamics

**Fig. 4:**
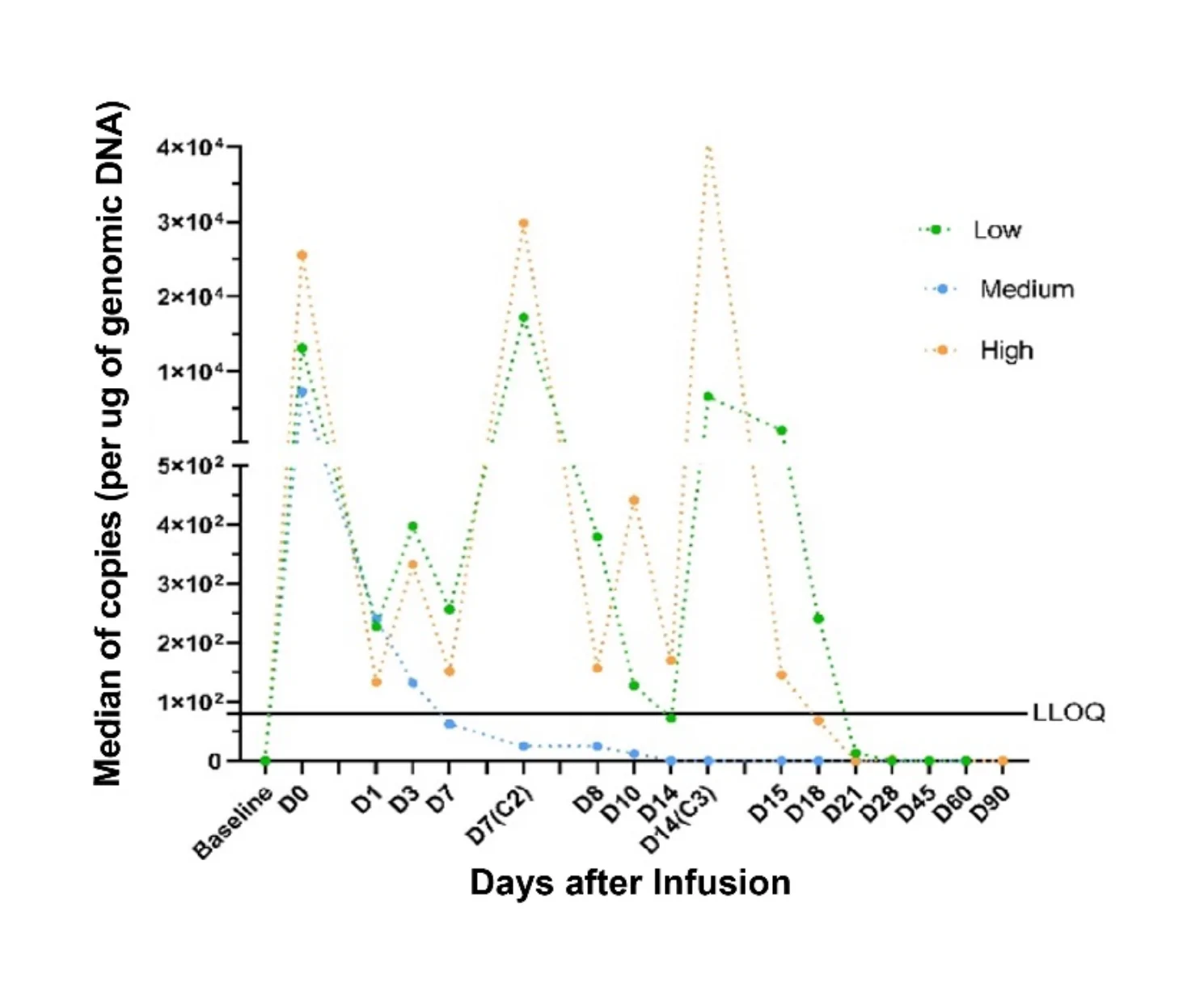
The persistence of CD33 CAR-NK cells in the peripheral blood of patients enrolled in the clinical study was evaluated using qPCR

CD33 CAR-NK cells could be observed by qPCR within 7 days. The peak concentration occurred 6 h after each infusion, followed by a second peak within two days of the initial peak (Fig. 4).

## Discussion

Compared with CAR-T-cell therapy, CD33 CAR-NK-cell therapy has a more favorable safety profile. For example, in clinical trials of CAR-T-cell therapy for ALL, the incidence of grade 3–4 cytokine release syndrome (CRS) is approximately 30%, while the incidence of ICANS is approximately 10% [14]; however, few severe AEs have been reported in CAR-NK-related clinical trials. In CAR-T-cell therapy targeting TAAs in AML, the most challenging AE is severe and prolonged bone marrow suppression, which has been fatal in several reported trials. Allo-HSCT is required to eliminate residual CAR-T cells and alleviate bone marrow suppression caused by on-target, off-tumor effects [15]. CD33 CAR-NK cell therapy prevents the side effects of the modest proliferative capacity of NK cells. Previous research in a mouse model revealed that there was no HSCT damage after CAR-NK-cell infusion.

CD33 CAR-NK cell therapy prevents the side effects associated with the limited proliferative capacity of NK cells. In previous studies in a mouse model, no HSCT damage was observed following CAR-NK cell infusion. Considering the existing CAR-T-cell and CAR-NK-cell trials, we designed a two-stage exploratory trial that allowed patients to receive infusions from 6 × 10^8^ cells, which is the maximum dose of CAR-T-cell therapy, up to 1.8 × 10^9^ cells, which is intermediate in the range of reported CAR-NK-cell therapy doses, at intervals of 7 days. None of the 3 patients in phase one experienced any dose-limiting toxicity, and each patient received 1.8 × 10^9^ CD33 CAR-NK cells. After determining that 1.8 × 10^9^ cells is a safe dose, we further recruited 3 patients who received one dose of 1.8 × 10^9^ cells and 4 patients who received 3 doses of 1.8 × 10^9^ cells. Only one patient experienced grade 2 CRS, which was alleviated with a 5 mg dose of intravenous dexamethasone. All cases of fever were self-limiting and resolved with supportive care. Additionally, all instances of severe bone marrow suppression resolved within one month, highlighting the favorable safety profile of the treatment. These results indicate that the well clinical tolerance of CD33 CAR-NK cells in treating R/R AML.

In terms of efficacy, newly introduced agents for AML treatment have shown limited effectiveness against R/R AML, with a CR rate of approximately 30% and a typical relapse occurring within six months [16]. Our research has demonstrated the safety and preliminary efficacy of CD33 CAR-NK cell therapy in the treatment of R/R AML, showing its potential to induce complete remission. However, similar to CAR-T-cell therapy, recurrent disease remains the primary cause of mortality. Owing to the limited expansion capacity of CD33 CAR-NK cells, long-term survival was achieved in only one patient. We hypothesized that repeated infusions of CD33 CAR-NK cells would prolong remission and increase the antitumor efficacy. However, after multiple infusions of 1.8 × 10⁹ cells, no significant increase in efficacy was observed, likely due to patient heterogeneity. Additionally, the recovery of the immune system may lead to the elimination of CAR-NK cells following infusion, as evidenced by the diminishing peaks observed after each dose. These findings suggest that the current design may not be optimal for CAR-NK cell therapy. Compared with the reported results of induced pluripotent stem cell (iPSC)-induced CD33 CAR-NK cell therapy, the safety and efficacy of umbilical cord-derived CD33 CAR-NK cells appear to be comparable [17]. The limitation of efficacy in this umbilical CD33 CAR-NK cell therapy is the limited long-term remission time after remission, which may be caused by the limited persistence of CD33 CAR NK cells. The possible reason could be the results combining inter-donor variability, the character of NK cells, and the influence of AML microenvironment. The AML microenvironment and heave tumor burden could be the reasons for limited persistence compared with CD19 CAR-NK cells published.

## Conclusion

In this trial, we demonstrated the safety and efficacy of CAR-NK cell therapy. On the basis of these findings and advancements in preclinical methods, CAR-NK cells could be further explored as potential treatments for AML. However, increases in persistence and efficacy are still needed, particularly through the use of advanced cytokines to support NK cell function and prevent depression [18–21].

## Electronic supplementary material

Below is the link to the electronic supplementary material.

Supplementary Material 1

Supplementary Material 2

## Data Availability

No datasets were generated or analysed during the current study.

## Abbreviations

AEs: Adverse events
ALL: Acute lymphoblastic leukemia
allo-HSCT: Allogeneic HSCT
AML: Acute myeloid leukemia
B-ALL: B-cell acute lymphoblastic leukemia
CAR: Chimeric antigen receptor
CBMCs: Cord blood mononuclear cells
CRS: Cytokine release syndrome
GVHD: Graft-versus-host disease
HLA: Human leukocyte antigen
HSCs: Hematopoietic stem cells
HSCT: Hematopoietic stem cell transplantation
ICANS: Immune effector cell–associated neurotoxicity syndrome
iCR: Imaging complete response
ICU: Intensive care unit
iPSC: Induced pluripotent stem cell
IWG: International Working Group
MRD: Minimal residual disease
MRD-CR: Minimal residual disease–negative complete response
NK: Natural killer
OS: Overall survival
PFS: Progression-free survival
R/R: Refractory or relapsed
R/R: AML relapsed/refractory acute myeloid leukemia
TAA: Tumor-associated antigen
TSA: Tumor-specific antigen
