# Correction: Safety and efficacy of CD33-targeted CAR-NK cell therapy for relapsed/refractory AML: preclinical evaluation and phase I trial

**DOI:** 10.1186/s40164-026-00841-w

**Published:** 2026-10-26

**Authors:** Ruihao Huang, Xiaoqi Wang, Hongju Yan, Xu Tan, Yingying Ma, Maihong Wang, Xiao Han, Jia Liu, Li Gao, Lei Gao, Guangjun Jing, Cheng Zhang, Qin Wen, Xi Zhang

**Affiliations:** 1 Medical Center of Hematology, Xinqiao Hospital of Army Medical University, Chongqing, 400037 China; 2 Chongqing Key Laboratory of Hematology and Microenvironment, Chongqing, 400037 China; 3 State Key Laboratory of Trauma and Chemical Poisoning, Army Medical University, Chongqing, 400037 China; 4 iCareab Biotechnology Co., Ltd., Shanghai, China; 5 Jinfeng Laboratory, Chongqing, 401329 China

**Correction: Experimental Hematology & Oncology (2025) 14:1**

**https://doi.org/10.1186/s40164-024-00592-6**

Following the publication of this article [1], the authors noticed the errors in Materials and Methods section, Supplementary Material 2 and panels of Fig. 2B and C. The incorrect and correct versions have been presented with this correction.

In Materials and Methods section, the mice “B6129SF1/J” was inadvertently used instead of “NSG” in the first paragraph of “In vivo animal model” section. The sentence should read “NSG mice received 1.5 × 10^6 CAR-T or 1 × 10^7 CAR-NK cells via the tail vein” instead of “B6129SF1/J mice received 1.5 × 10^6 CAR-T or 1 × 10^7 CAR-NK”.

In Fig. 2, the corresponding P values are missing in the image of Fig. 2B and C.

**Incorrect Fig. 2:**

**Figure:**
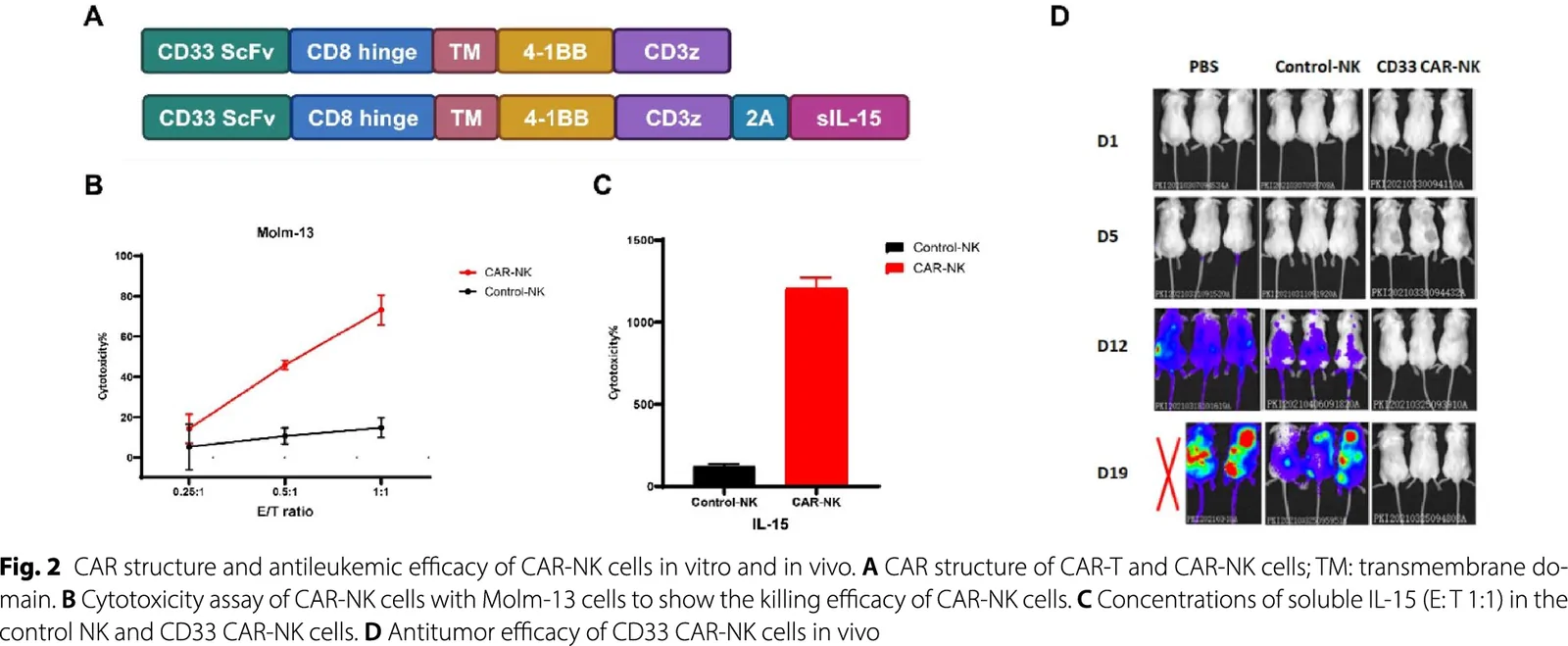

**Corrected Fig. 2:**

**Fig. 2:**
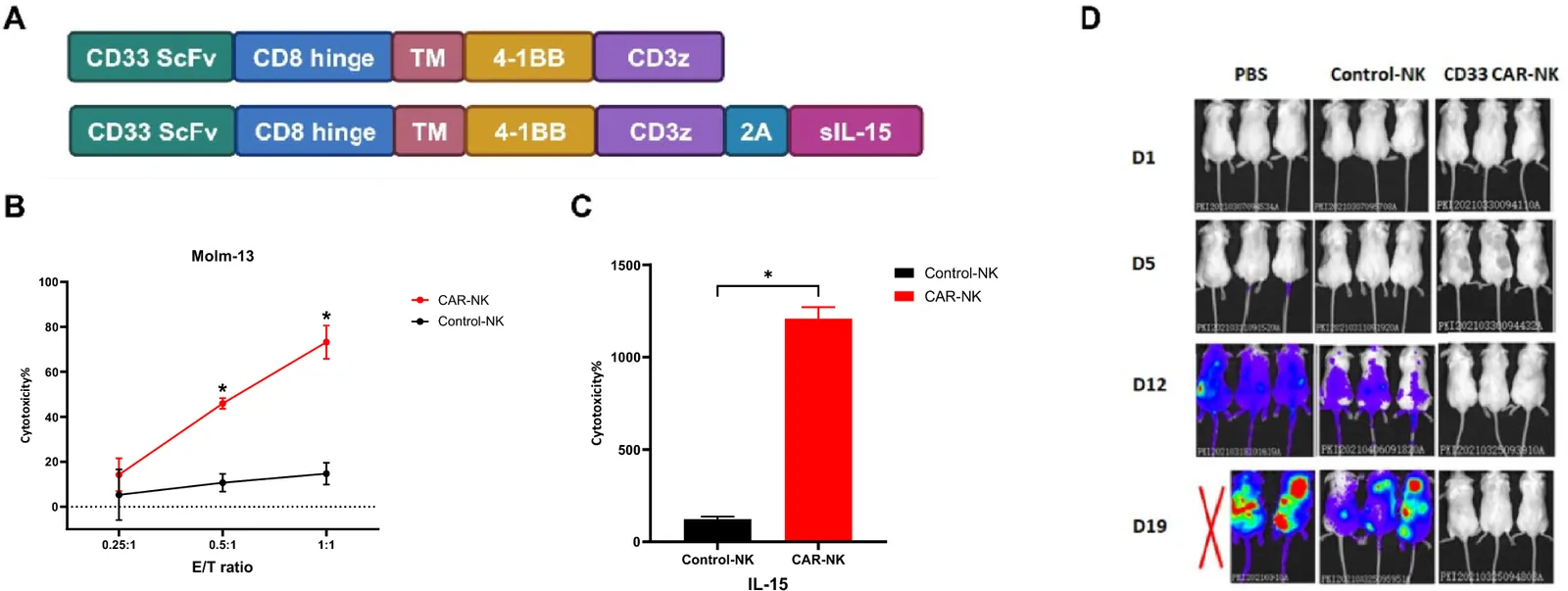
CAR structure and antileukemic efficacy of CAR-NK cells in vitro and in vivo. **A** CAR structure of CAR-T and CAR-NK cells; TM: transmembrane domain. **B** Cytotoxicity assay of CAR-NK cells with Molm-13 cells to show the killing efficacy of CAR-NK cells. **C** Concentrations of soluble IL-15 (E: T 1:1) in the control NK and CD33 CAR-NK cells. **D** Antitumor efficacy of CD33 CAR-NK cells in vivo

In Supplementary Material 2, the doses used in the study are consistent with those specified in the registered clinical trial. The wrong version of Supplementary Material 2 has now been replaced with the correct file.

## Supplementary Information

Supplementary Material 2. CLINICAL TRIAL PROTOCOL.

## References

[1] Huang R, Wang X, Yan H, Tan X, Ma Y, Wang M, et al. Safety and efficacy of CD33-targeted CAR-NK cell therapy for relapsed/refractory AML: preclinical evaluation and phase I trial. Exp Hematol Oncol. 2025;14:1. 10.1186/s40164-024-00592-6. PMC11694373 39748428

