# Supplementary material for: Safety and efficacy of CD33-targeted CAR-NK cell therapy for relapsed/refractory AML: preclinical evaluation and phase I trial
Source: Exp Hematol Oncol. 2025 Jan 2;14:1. doi: 10.1186/s40164-024-00592-6 (PMC11694373; doi:10.1186/s40164-024-00592-6)
Supplement: Supplementary file 1 — Supplementary Material 1 [file 40164_2024_592_MOESM1_ESM.pdf]

Supplementary Information

Safety and Efficacy of CD33- targeted CAR-NK Cell Therapy for Relapsed/Refractory AML: Preclinical Evaluation and Phase I trial

Ruihao Huang, Xiaoqi Wang, Hongju Yan, Xu Tan, Yingying Ma, Maihong Wang, Xiao Han, Jia Liu, Li Gao, Lei Gao, Guangjun Jing, Cheng Zhang, Qin Wen, Xi Zhang

Supplement 1.

<u>First Activation of CAR-NK</u>

Reagent Preparation

HPBS Solution: Mix HSA solution and DPBS solution at a 1:19 (v/v) ratio.

Med NK Medium: Add 2.5% SUPERGROW cell culture supplement, and finally add IL-2 solution to achieve a final concentration of 500 IU/ml.

Thawing and Counting of CBMC Cells

Prepare a 50 ml centrifuge tube with 10–20 ml Med NK medium. Transfer thawed cells from the cryopreserved vial or bag to the centrifuge tube.

Balance the centrifuge and centrifuge at 22°C, 500 g for 5 minutes, with an acceleration of 8 and deceleration of 5. If the cell suspension volume is ≥30 ml, centrifuge for 10 minutes. Discard the supernatant, add 1-10 ml HPBS, and mix well. Count and record cell density and viability using a cell counter.

Washing Magnetic Beads

Calculate T cell quantity = CBMC cell count × CD3+ percentage.

Add magnetic beads at a 1:1 ratio to the T cell quantity. Bead volume (μl) = (T cell count/1E+08)× 1000 μl.

Add 0.5–1 ml HPBS to the cryopreserved tube, mix with the required volume of magnetic beads, place in a magnetic rack for 2 minutes, discard the supernatant, add 0.2–1 ml HPBS, remove from the rack, and mix well.

Co-incubation of Cells with Magnetic Beads

Adjust the density of the cell suspension to 0.1–1×10^8 cells/ml with HPBS, and add DNase I at a ratio of 3 μl per 10 ml.

Add the magnetic bead suspension to the cell suspension, mix well, and incubate at room temperature for 30 minutes with shaking。

Sorting and Removing T Cells to Obtain CD3-Negative CBMC Cells

Insert the centrifuge tube containing the cell and magnetic bead mixture into a magnetic rack for 2 minutes. Using a pipette, transfer the supernatant to another new centrifuge tube. The cells in this supernatant are the T-cell depleted CD3 negative CBMC.

Remove the centrifuge tube from the magnetic rack, add 1-10 ml HPBS to resuspend the cells, and then reinsert the tube into the magnetic rack for 2 minutes. Using a pipette, transfer the supernatant and combine it into the same centrifuge tube.

Balance the centrifuge tube containing the CD3-negative CBMC cells and centrifuge at 300 g for 5 minutes with an acceleration of 8, deceleration of 5, at 22℃. (If the cell suspension in the centrifuge tube is ≥30 ml, centrifuge for 10 minutes).

After centrifugation, discard the supernatant. Add 1-10 ml Med NK medium to resuspend the cells. Transfer 20-50 μl of the cell suspension to a 1.5 ml EP tube, and count and record the cell density and viability.

Based on the counting results, take more than 1×10^6 cells for CD3+ and CD56+ testing. Transfer the remaining cell suspension to a T75/T175 flask, add Med NK medium, and adjust the cell density to 1.5-2×10^6 cells/ml. Place the culture flask in a CO2 incubator at 37°C with 5% CO2 for incubation.

Thawing Feeder Cells

Calculate the required number of feeder cells to be thawed according to the ratio of CD3-negative CBMC cells to feeder cells, which is 1:1 (a ratio within the range of 1:1 to 1:1.2 is acceptable). Based on the number of feeder cells needed, select the appropriate number and size of vials (A1/A2) for thawing.

Prepare a 50 ml centrifuge tube, and add 10–20 ml of Med NK medium to the tube. Transfer the thawed feeder cells into the centrifuge tube.

Balance the centrifuge tube and centrifuge at 22℃, 300 g for 5 minutes, with an acceleration of 8 and deceleration of 5. (If the cell suspension volume in the centrifuge tube is ≥30 ml, centrifuge for 10 minutes).

After centrifugation, discard the supernatant. Add 10–30 ml of Med NK medium, resuspend the cells by pipetting to mix thoroughly. Transfer 20–50 μl of the cell suspension to a 1.5 ml EP tube, and count and record the cell density and viability.

NK Cell Activation

Add the feeder cells to the CD3-negative CBMC cell culture flask at a ratio of CD3-negative CBMC to feeder cells equal to 1:1 (a ratio within the range of 1:1 to 1:1.2 is acceptable). Adjust the cell density to 1.5–2.5×10^6 cells/ml by adding Med NK medium

according to the total number of CD3-negative CBMC and feeder cells. Place the culture flask in a CO2 incubator (37°C, 5% CO2) for incubation.

Observation and Supplementation of NK Cells (Day 1–Day 3)

On Day 1, Day 2, and Day 3, take out the culture flask and observe under a microscope to check for cell clustering. If the culture medium turns yellow and the cell density significantly increases, supplement the medium directly by adding 1/4 to 1/2 of the volume. Perform the supplementation operation gently. Place the flask back into the CO2 incubator (37°C, 5% CO2) for continued incubation.

Virus Transduction Operation (Day 4)

Take out the culture flask and gently shake it. Using a pipette, transfer the cell suspension to a 50 ml centrifuge tube, balance it, and centrifuge. Centrifuge conditions: 22°C, 300g for 5 minutes, acceleration 8, deceleration 5. (If the cell suspension volume in the centrifuge tube is ≥30 ml, centrifuge for 10 minutes).

After centrifugation, discard the supernatant. Using a pipette, add 10–30 ml of Med NK medium to resuspend the cells. Transfer 20–50 µl of the cell suspension to count and record cell density and viability.

Take the number of cells to be transduced and adjust the cell density to 2×10^6 cells/ml with Med NK medium, resulting in a total volume of V1. Adjust the remaining cells to a density of 1–1.5×10^6 cells/ml for the blank NK culture.

Transfer the cell suspension to a T75/T175 culture flask and incubate in a CO2 incubator (37°C, 5% CO2) for at least 1 hour.

Calculate the required virus volume (V_virus) based on the number of cells to be transduced, the multiplicity of infection (MOI), and the virus titer. Take the lentivirus out of the -80°C freezer and place it in a 4°C refrigerator for thawing 10-30 minutes prior to use.

According to the number of cells to be transduced, take out the T75/T175 culture flask, pipette the volume of Med NK medium V2 (V1 - V_virus - V_adjuvant) and add it to the culture flask; pipette the virus volume V_virus and add it to the culture flask, mix well; pipette the required NovoNectin solution volume V_adjuvant (working concentration 5 µg/ml) and add it to the culture flask, mix well. Incubate in the

incubator (37°C, 5% CO2) for 30 minutes.

After the incubation of NK cells with the lentivirus, add the NK cell suspension to the culture flask containing the lentivirus, mix thoroughly. Place the flask in the CO2 incubator (37°C, 5% CO2) for incubation.

Centrifugation and Medium Exchange (Day 5)

After culturing for 20-26 hours, take out the culture flask. Using a pipette, transfer the cell suspension to a 50 ml centrifuge tube, balance it, and centrifuge. Centrifuge conditions: 22°C, 300 g for 5 minutes, acceleration 8, deceleration 5. (If the cell suspension volume in the centrifuge tube is ≥30 ml, centrifuge for 10 minutes).

After centrifugation, discard the supernatant. Using a pipette, add 10-30 ml of Med NK medium to resuspend the cells. Transfer 20-50 μl of the cell suspension to a 1.5 ml EP tube, and count and record cell density and viability.

Transfer the cell suspension to a T75/T175 culture flask, add Med NK medium to adjust the cell density to 1-2×10^6 cells/ml, and place the flask in a CO2 incubator (37°C, 5% CO2) for incubation.

On Day 6, take out the culture flask and observe the cells under a microscope to check for cell clustering and normal cell status. If the medium turns yellow or the cell density significantly increases, supplement the medium directly by adding 1/2 to 1 times the volume, and place the flask back into the CO2 incubator for continued incubation.

<u>CAR-NK Second Activation</u>

Reagent Preparation

Med NK Medium Preparation: Add 2.5% Dakovirus SUPERGROW cell culture additive to KBM 581 medium. Finally, add IL-2 solution to achieve a final IL-2 concentration of 500 IU/ml.

Sample Counting

Culture the cells until Day 7, then take out the culture flask. Mix the cell suspension in the culture flask thoroughly, and take 20-50 μl of the cell suspension for cell density and viability measurement using a cell counter. After sampling, place the culture flask back in the incubator for continued incubation.

Based on the counting results, take more than 1×10^6 cells for testing CD3+, CD56+, and CAR+。

If flow cytometry detects CD3+ ≥ 1%, negative selection is required; if CD3+ < 1%, negative selection is not necessary.

Feeder Cell Thawing

Calculate the required number of feeder cells to be thawed according to the ratio of NK cells to feeder cells, which is 1:1 (a ratio within the range of 1:1 to 1:1.2 is acceptable). Based on the number of feeder cells needed, select the appropriate number and size of vials (A1/A2) for thawing.

Prepare a 50 ml centrifuge tube, and add 10-20 ml of Med NK medium to the tube. Transfer the thawed feeder cells into the centrifuge tube.

Balance the centrifuge tube and centrifuge at 22℃, 300 g for 5 minutes, with an acceleration of 8 and deceleration of 5. (If the cell suspension volume in the centrifuge tube is ≥30 ml, centrifuge for 10 minutes).

After centrifugation, discard the supernatant. Add 10-30 ml of Med NK medium, resuspend the cells by pipetting to mix thoroughly. Transfer 20-50 µl of the cell suspension to a 1.5 ml EP tube, transfer to a class C counter, and record cell density and viability.

CAR-NK Activation

Add feeder cells to the cell culture flask at a ratio of NK cells to feeder cells equal to 1:1 (a ratio within the range of 1:1 to 1:1.2 is acceptable). Adjust the cell density to 1.0-2.0×10^6 cells/ml by adding Med NK medium according to the total number of NK and feeder cells. Mix thoroughly and place the culture flask in a CO2 incubator (37°C, 5% CO2) for incubation.

<u>**CAR-NK Expansion**</u>

Reagent Preparation

Med NK Medium Preparation: Add 2.5% Dakovirus SUPERGROW cell culture additive to KBM 581 medium. Finally, add IL-2 solution to achieve a final IL-2 concentration of 500 IU/ml.

Cell Counting

Culture the cells until Day 9, then take out the culture flask. Mix the cell suspension in the culture flask thoroughly, and take 20-50 μl of the cell suspension for cell density and viability measurement using a cell counter.

Cell Supplementation

Based on the counting results, determine the volume of supplementation needed and whether subculturing (splitting) is necessary. Adjust the cell culture density to 1.0-2.0×10^6 cells/ml.

Counting and Supplementation

When the culture volume in G-Rex100 or ImmunoVessel-500 is less than 400 ml, perform cell counting and supplementation every 1-3 days (for sampling counts, either count all flasks or randomly select half of the flasks for sampling counts). Based on the counting results, determine whether subculturing is necessary and adjust the cell culture density to $1.0\text{-}2.0\times10^6$ cells/ml. When the culture volume in G-Rex100 or ImmunoVessel-500 reaches 400 ml, perform cell counting and medium exchange every 1-2 days, with an exchange volume of 200-280 ml.

<u>CAR-NK Cryopreserved Preparation</u>

Reagent Preparation

WS1: Prepare Wash buffer WS1 in the ratio of Compound Electrolyte Injection to Human Albumin 19:1.

WS2: Wash buffer WS2 is prepared in the ratio of compound electrolyte injection to human albumin 1:3.

Cell Counting

Take out the G-Rex10 or ImmunoVessel-5000 culture flasks from the CO2 incubator under the static culture state, remove 200~280 ml of supernatant in the flasks, blow and mix the remaining cell suspension, and take samples for counting.

Sample testing

According to the counting results, take more than 1.0×106 cells in EP tubes, and detect CD3+, CD56+, CAR+. et al.

Cell Harvesting

When the total number of cells reaches the desired number of cells, the culture can be stopped and frozen preparations can be carried out. Transfer the remaining cell suspension of the culture flask to the centrifuge tube, leveling centrifugation, centrifugation conditions: 22°C, 500g, 10 min, with an acceleration of 8, deceleration of 5.

First washing: remove the supernatant at the end of centrifugation. Resuspend the cells with WS1 and combine the cell suspensions in 3-5 centrifuge tubes and centrifuge flat. Centrifugation conditions: 22°C, 500g, 10 min, with an acceleration of 8, deceleration of 5.

Second wash: remove the supernatant at the end of centrifugation. Resuspend the cells with WS1 and combine the cell suspensions from 3-5 tubes and centrifuge flat. Centrifugation conditions: 22°C, 500g, 10 min, with an acceleration of 8, deceleration of 5.

At the end of centrifugation, remove the supernatant, add WS2 to resuspend the cells and combine to 1 tube, mix well. Take 20~50 μl of cell dilution suspension (diluted with Med NK medium), and detect cell density and viability by cell counter.。

Mycoplasma sampling: 2 ml WS2 in a sterile heatless centrifuge tube, and according to the counting results of ≥2E+06 cells in the centrifuge tube, sealing film sealing, sent for QC.

Freezing preparation dispensing

According to the required cell number and preparation density (1.0~5.0×10^7 cells/ml) calculated above. Take the required volume of V cells and VWS2 with a pipette and mix well, then make up the corresponding volume of VIL2 and mix well while adding VCS10. Select the appropriate product bag and dispense. Heat-seal the line after removing air bubbles from the product bag, and check the sealing effect and label the product. Take the cell suspension of QR QC and dispense it into cryopreservation tubes and label it. Freeze storage after preparation is completed.

Sample testing

Endotoxin: take 1 ml of cell suspension in a sterile and heat-free freezing tube, seal

with sealing film and send for QC.

Asepsis: take at least 5 ml of cell suspension in a sterile heatless centrifuge tube with a pipette, seal with a sealing film and send for QC.

Supplement 2. CD33 CAR-T manufacture

On day 0, thaw PBMCs and add 1 μL of Universal Nuclease to a 10 mL culture system, allowing it to stand for 3 hours. Perform CD3 positive selection by adding Dynabeads at 15% of the MT-14 volume, and culture the cells at 2×10^6/mL, monitoring the bead-to-cell ratio under a microscope. On day 2, observe activated T cells, then collect and centrifuge them at 300g for 5 minutes. Resuspend and count the cells in X-VIVO15+5% FBS medium containing an IL mix, and culture them at 2×10^6/mL for 1 hour. Take 1 mL of T cells (2×10^6/mL), add 1 mL of X-VIVO+5% FBS medium with 10 μg/mL DEAE, then add 200 μL of virus, mix well, and centrifuge at 800g for 1.5 hours. After centrifugation, gently tap the tube and continue culturing the cells. From days 3 to 7, centrifuge the cells at 300g for 5 minutes and refresh the medium. On day 5, remove the magnetic beads before centrifugation, and perform a quality check on day 7.

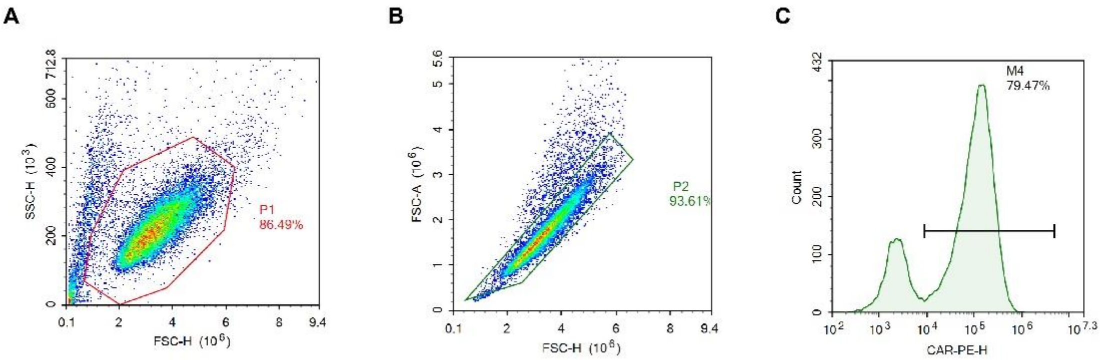

Supplemental Figure . CD33 CAR-T manufacture

Supplement 3. Selection of anti-CD33 optimal single-chain

To evaluate the activation and proliferative capacity of CD33 CAR-T cells, we co-cultured these cells with Molm13 cells. The levels of IL-2, IFN-γ, and TNF-α were measured in the supernatant 24 hours after co-culture to assess CAR-T cell activation. Additionally, CAR-T cell proliferation was quantified after 7 days. Both the 1 and 2 CAR constructs demonstrated superior efficacy in vitro compared to the control group, showing enhanced activation and proliferation.

To further validate these findings in vivo, we established an AML model by injecting Molm13-luc cells into NSG mice. Seven days after tumor engraftment, the mice were treated with 1.5×10^6 CAR-T cells administered intravenously. T cells expressing either the 1 or 2 CAR constructs exhibited a significant antitumor response in the AML model, as evidenced by reduced tumor burden compared to both the negative control group and those receiving the conventional CD33 CAR construct.

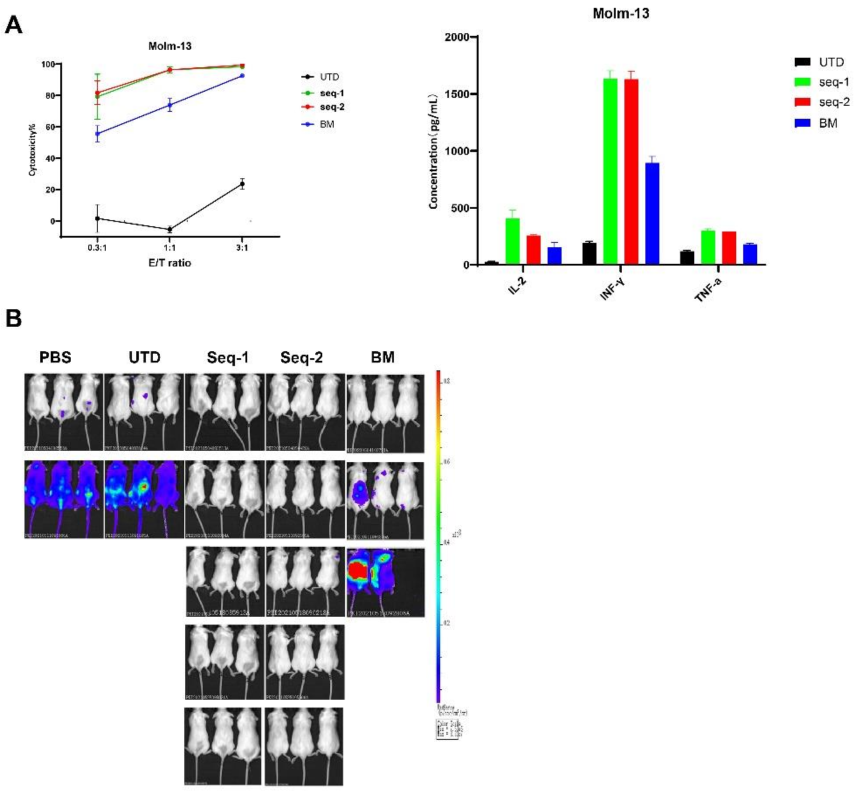

Supplemental Figure . Selection of anti-CD33 optimal single-chain

Supplement 4. Hematopoietic Stem Cell Safety

To assess the toxicity of CAR-T cells on HSCs, an *in vitro* hematopoietic progenitor CFU assay was conducted. Thawed CD34 cells were diluted to 5×10^4/mL in IMDM+2% FBS, and CAR-positive CAR-T cells were added at a 10:1 ratio, with blank T cells adjusted to ensure consistent total T cell counts across groups. A 100 μL mixture of CAR-T and CD34 cells was incubated in a 24-well plate for 4 hours, then combined with MethoCult™ medium and dispensed into culture dishes. These were incubated at 37°C with 5% CO_2 for 14-16 days, with periodic checks to ensure proper humidity. Colony formation (BFU-E, CFU-G, CFU-GEMM) was analyzed based on growth morphology.

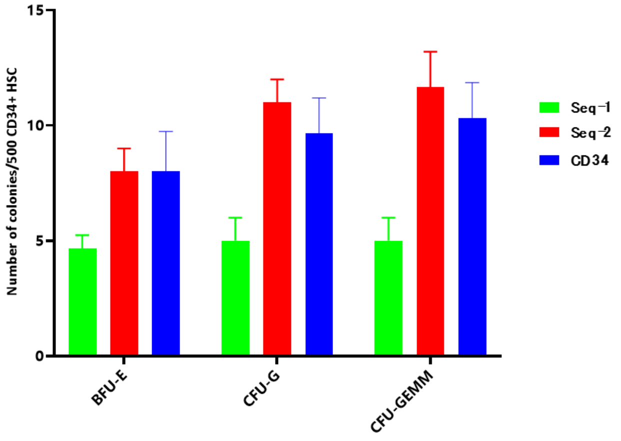

Supplemental Figure The toxicity of CAR-T cells on HSCs
