# Supplementary material for: Safety and efficacy of CD33-targeted CAR-NK cell therapy for relapsed/refractory AML: preclinical evaluation and phase I trial
Source: Exp Hematol Oncol. 2025 Jan 2;14:1. doi: 10.1186/s40164-024-00592-6 (PMC11694373; doi:10.1186/s40164-024-00592-6)
Supplement: Supplementary file 2 — Supplementary Material 2 [file 40164_2024_592_MOESM2_ESM.pdf]

Prospective Clinical Study of the Safety and Efficacy of Anti-CD33 CAR-NK Cells in the Treatment of Relapsed/Refractory Acute Myeloid Leukemia

Version date	:	12/01/2021
Institution	:	The Second Affiliated Hospital of Army Medical University (Xinqiao Hospital of Army Medical University)

BACKGROUND

Acute Myeloid Leukemia (AML) is a common hematologic malignancy that originates from the malignant proliferation of hematopoietic stem/progenitor cells (HSPCs) in the hematopoietic system. This malignancy primarily arises from genetic alterations that disrupt the differentiation and maturation of myeloid cells and inhibit apoptosis, leading to their uncontrolled proliferation and accumulation in the bone marrow, ultimately impairing normal hematopoiesis. AML accounts for approximately 70% of all acute leukemias, with an incidence rate of 1.6 to 2.3 per 100,000 population, and its incidence is increasing yearly. It can occur at any age, with a sudden onset and rapid progression, leading to a natural survival period of 6 months to 1 year. AML has become a life-threatening disease with severe implications for human health.

Conventional treatments, such as chemotherapy and hematopoietic stem cell transplantation (HSCT), achieve a cure rate of 35% to 40% in adults under the age of 60, but the cure rate drops to only 5% to 15% for patients over 60. Among patients who fail to achieve remission with induction therapy, the 5-year survival rate is merely 7% to 12%. Statistics show that 50% to 80% of patients who achieve complete remission (CR) eventually relapse, progressing to refractory leukemia and ultimately succumbing to the disease. Although the introduction of combination chemotherapy, HSCT, targeted therapies, and cellular therapies has significantly improved remission rates compared to the past, overall long-term survival remains below expectations. Chemotherapy resistance and early relapse following remission continue to be major challenges in AML treatment. CAR-T cell therapy has ushered in a new era of cellular therapy, with advances in gene editing technologies further enhancing the anti-tumor efficacy of cell-based treatments. In the treatment of hematologic malignancies, immune cell therapies, particularly CAR-T cell therapy, have proven to be effective in inducing remission. In certain cases, these therapies have even succeeded in rescuing patients who have not responded to HSCT. Natural Killer (NK) cells play a crucial role in the graft-versus-leukemia (GVL) response and are vital in preventing relapse after HSCT. NK cells eliminate tumor cells through several mechanisms, including the secretion of cytotoxic granules, expression of tumor necrosis factors, mediation of antibody-dependent cell-

mediated cytotoxicity (ADCC), and the release of cytokines. Chimeric Antigen Receptor (CAR)-NK cell immunotherapy is a form of adoptive cellular immunotherapy against tumors. Gene-engineered NK cells combine specific tumor antibody recognition with co-stimulatory signaling, enabling tumor antigen-specific recognition, proliferation, and cytotoxicity independent of the major histocompatibility complex (MHC). In recent years, the advantages of CAR-NK cell therapy have become increasingly apparent. Unlike T cells, NK cells do not require gene editing to be allogeneic and can be produced as off-the-shelf products, which saves production time and costs. Furthermore, NK cells do not induce graft-versus-host disease (GvHD), and they do not secrete inflammatory cytokines, such as IL-1 and IL-6, which are associated with cytokine release syndrome (CRS), making them safer than T cells.

CD33 is a myeloid-specific antigen expressed in hematopoietic cell subsets. It is expressed at low levels on normal hematopoietic stem/progenitor cells but is highly expressed in approximately 90% of AML patients. CD33 is primarily targeted in AML therapies. According to clinical studies on a similar product, TAK-007 (Takeda/MD Anderson Cancer Center, CD19 CAR-NK, umbilical cord blood-derived), NK cells derived from umbilical cord blood can be expanded using feeder layer methods for 14 days, achieving a 3,127-fold expansion with an NK cell purity of over 80%. Preclinical studies of TAK-007 have demonstrated potent anti-tumor activity, and clinical trials have yielded positive results, with an objective response rate (ORR) of 73% and a complete remission (CR) rate of 64% in 11 enrolled patients with hematologic malignancies. The treatment was well-tolerated, with no cases of CRS, neurotoxicity, GvHD, or significant increases in inflammatory cytokines. The main Grade 3/4 adverse events were hematologic toxicity. In this study, we utilized a CD33-targeting CAR-NK cell therapy product developed by the Hematology Medical Center at the Army Medical University for the treatment of relapsed/refractory AML. The product employs a CAR structure designed with a single-chain antibody fragment (scFv) targeting the CD33 antigen, with both in vitro and in vivo experiments confirming the CAR-NK cells' cytotoxicity against CD33+ cells.

This product is an umbilical cord blood-derived NK cell preparation engineered in vitro

using lentiviral modification. The modified NK cells express CAR molecules on their surface and secrete IL-15 to enhance the survival and proliferation of CAR-NK cells in vivo. The CAR molecule uses a humanized antibody sequence to recognize the CD33 molecule on the cell surface. Through the 4-1BB and CD3ζ signaling domains in the cytoplasmic region, activation signals are transmitted to the NK cells. Upon full activation, NK cells secrete effector molecules and IFN-γ to kill tumor cells.

PRUPOSE

Primary objectives:

1. To assess the safety of anti-CD33 CAR-NK cells for the treatment of relapsed, refractory acute myeloid leukemia (AML) and to observe dose-limiting toxicity (DLT);
2. Determine the maximum tolerated dose (MTD), and/or the recommended phase I dose (RP1D).

Secondary objective:

1. To assess the preliminary efficacy of anti-CD33 CAR-NK cells in the treatment of relapsed, refractory AML;
2. To assess the in vivo cellular metabolic kinetics (PK) and immunogenicity of anti-CD33 CAR-NK cells.

RESEARCH DESIGN

1. Overall design

This study used an open, single-arm study design to evaluate the safety and preliminary efficacy of anti-CD33 CAR-NK cells in the treatment of relapsed, refractory AML.

2. Inclusion and exclusion of participants

This study is being conducted at the Hematology Medical Center, Second Affiliated Hospital of Army Medical University. The criteria for participant inclusion include patients diagnosed with AML who have a limited overall survival despite existing therapies. The specific requirements are as follows.

Inclusion Criteria

(1)Chinese participants aged ≥18 and ≤70 years at the time of signing the informed consent form;

(2)ECOG performance status of 0 or 1, with an expected survival of ≥3 months;
(3)Patients meeting any of the following conditions: ① Acute myeloid leukemia (excluding M3) that has not achieved complete remission after standard chemotherapy regimens; ② AML patients who do not qualify for, lack access to, or refuse allogeneic hematopoietic stem cell transplantation (HSCT);
(4)CD33 expression on malignant cells must be detected by immunohistochemistry or flow cytometry;
(5)Clinical laboratory values during the screening period must meet the following criteria: ① Creatinine ≤2.5 times the upper limit of normal (ULN); ② Baseline oxygen saturation ≥90%; ③ Total bilirubin ≤3 times ULN; ④ ALT and AST ≤ 3 times ULN; ⑤ Hemoglobin ≥80g/L;
(6)No anti-cancer treatments, including chemotherapy, radiotherapy, immunotherapy (e.g., immunosuppressive drugs/corticosteroids), within two weeks prior to signing the informed consent form;
(7)The patient must have a suitable venous access for mononuclear cell collection and no other contraindications;
(8)Female participants of childbearing potential must have a negative high-sensitivity serum pregnancy test (β-hCG) at screening and before receiving the first doses of cyclophosphamide and fludarabine. Participants of childbearing potential must use effective medical contraception (regardless of gender) from the time of informed consent until at least 12 months after receiving the anti-CD33 CAR-NK cell infusion. Specific contraceptive measures are listed in Appendix 4;
(9)After discussion by the expert panel, the patient's condition was analyzed, and the benefits of participating in the clinical trial were deemed to outweigh the risks based on the patient's general health status;
(10)Participants must provide informed consent by signing the informed consent form, indicating that they understand the purpose and procedures of the study, are willing to participate, and can comply with the study's prohibitions and restrictions.

Exclusion Criteria

(1)Acute promyelocytic leukemia (APL M3): t(15;17)(q22;q12); (PML/RARα) and variants;

(2)Prior hematopoietic stem cell transplantation (HSCT);

(3)Diagnosis or treatment of another malignancy within the past 5 years, with the exception of adequately treated cervical carcinoma in situ, basal or squamous cell skin carcinoma, or localized prostate carcinoma or ductal carcinoma in situ treated with curative intent;

(4)Presence of severe systemic disease: New York Heart Association (NYHA) Class III or IV congestive heart failure; cerebrovascular accident or myocardial infarction within 6 months before signing the informed consent form; or hemodynamically unstable arrhythmias; impaired cardiac function (LVEF $<50\%$) as assessed by echocardiography;

(5)Severe concurrent illness, such as active or uncontrolled infections, within 14 days before signing the informed consent form;

(6)Pregnant or breastfeeding women;

(7)Central nervous system (CNS) leukemic involvement or Grade 3 CNS disease (e.g., $\geqslant 5/\mu L$ white blood cells in cerebrospinal fluid (CSF), positive cytocentrifuged smear for blasts without traumatic lumbar puncture, or clinical symptoms of CNS leukemia such as cranial nerve palsy from active disease); participants with adequately treated CNS leukemia are eligible;

(8)Seropositive for human immunodeficiency virus (HIV); hepatitis B surface antigen-positive or HBV DNA above the detection limit of the assay; hepatitis C antibody-positive or HCV RNA above the detection limit of the assay; seropositive for syphilis antibody and reactive for rapid plasma reagin; or CMV DNA-positive;

(9)Known life-threatening allergic reaction to anti-CD33 CAR-NK cells or their excipients, including dimethyl sulfoxide (DMSO); or a known hypersensitivity to biologic macromolecules such as antibodies or cytokines;

(10)Contraindications to fludarabine or cyclophosphamide treatment;

(11)Systemic corticosteroid therapy at doses greater than 20mg/day of prednisone (or equivalent) within two weeks prior to lymphodepletion;

(12)History of alcohol dependence, drug abuse, or psychiatric disorders;

(13)Any other conditions deemed by the investigator to make participation in this study inappropriate.

Termination Criteria

(1)Lack of Efficacy: If the treatment method in the clinical trial fails to achieve the expected therapeutic effect or if the results fall below pre-set standards, the study may be prematurely terminated.

(2)Safety Concerns: If severe adverse effects or complications occur during the clinical trial and are directly related to the treatment method, the study may be terminated early to protect the safety of the patients.

(3)Ethical Issues: If ethical concerns arise during the design or implementation of the clinical trial, such as violations of ethical principles or infringement on patient rights, the study may be terminated.

(4)Patient Issues: If a patient refuses to continue treatment, the clinical protocol must also be terminated. Respecting the patient's autonomy is a fundamental principle of medical ethics. When a patient explicitly expresses a desire to discontinue treatment, the physician must respect their choice and engage in thorough communication and explanation with the patient or their legal representative.

3. Sample size

This study is intended to include 10-18 subjects with relapsed, refractory AML.

4.Research outline

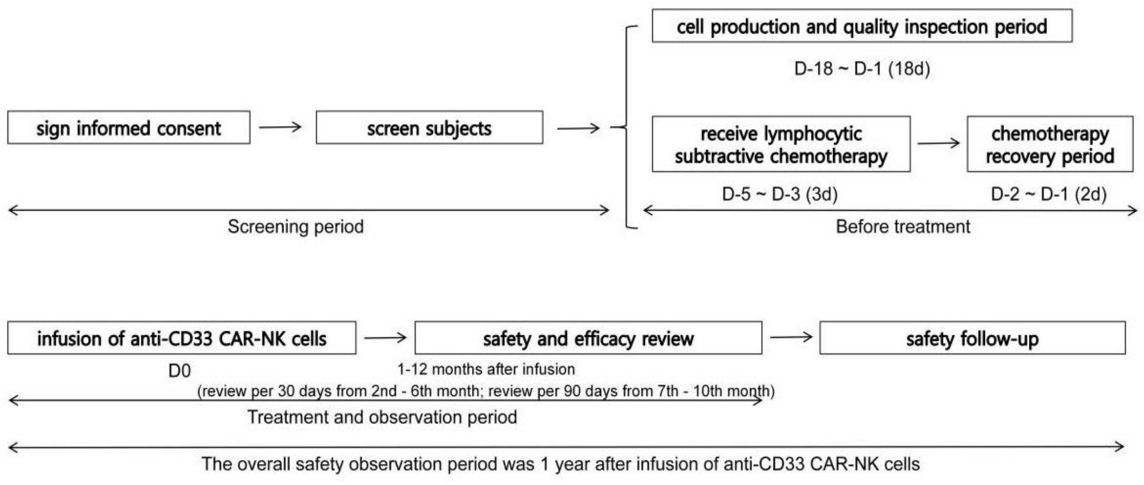

4.1 Participant Grouping and Methods

4.1.1 Screening (Day -38 to Day -18)

Investigators must thoroughly explain to each participant the nature, objectives, procedures, expected duration, potential risks and benefits, and any discomfort that may arise from the study during the informed consent process. Each participant must be informed that participation is voluntary, and they can withdraw from the study or revoke consent at any time without affecting their rights.

After explaining the basic aspects of the study and ensuring that the participants fully understand its purpose, the investigator should ask each participant or their legal guardian to sign, date, and provide contact information on the informed consent form. Before signing and dating, participants or their guardians should carefully read and consider the information. Upon understanding the study process and agreeing to participate, they should sign the informed consent form, which must also be signed by the investigator. Two copies of the consent form will be made: one will be kept by the national clinical research institution, and the other will be retained by the participant. Participants who have not provided informed consent or have not signed the informed consent form cannot participate in the study.

After signing the informed consent form, participants will undergo a screening medical examination and be assigned a "screening number" based on the order in which consent was signed. Participants are not allowed to undergo repeated screenings; however, if laboratory results do not meet the inclusion criteria, retesting within the screening window (Day -38 to Day -18) is permitted.

During the screening period, participants will undergo a comprehensive examination, and eligible participants will be selected. Demographic data and medical history will be recorded, along with assessments of disease consistency, height/weight measurements, vital signs, general physical examination, ECOG score, HLA typing, HLA antibodies, killer immunoglobulin-like receptors (KIR), complete blood count, blood chemistry, urinalysis, stool routine + occult blood, coagulation panel, cardiac enzyme profile, BNP, pregnancy test (for women of childbearing age), infectious disease screening, cytokine levels, ferritin, CRP, NK cell phenotype and function, cerebrospinal fluid examination, blood smear analysis, bone marrow aspiration and/or biopsy, bone marrow cytology, immunophenotyping, karyotype analysis (if necessary), FISH (if necessary), AML-related fusion genes (if necessary), AML-related gene mutations (if necessary), peripheral blood leukemic blasts, echocardiography,

electrocardiography, imaging assessment (if applicable), and health-related quality of life evaluation. At each visit, physical examinations, concomitant medications, and adverse events (AEs) will be recorded as required. The projects to be completed during the screening period are listed in the Table 1.

Table 1 Screening Items

List	Requirements and observation indicators
Demographic data	Date of birth, sex, nationality, age, nationality, etc
medical history	The medical history includes the current medical history (especially the tumor diagnostic information), previous disease history, medication history, allergy history, alcohol consumption history, drug abuse history, blood transfusion history, clinical trial history, previous surgery history, etc
Assessment of disease consistency	Evaluation of subjects diagnosed with relapsed and refractory acute myeloid leukemia (according to Chinese Guidelines for Diagnosis and Treatment of relapsed and refractory acute myeloid leukemia (2017 edition) (see Annex 7 for details)
Height / weight	\
vital sign	Respiratory rate, pulse, blood pressure, oxygen saturation, body temperature
General physical examination	Including the skin, mucous membrane, lymph nodes, head, neck, chest, abdomen, spine / limbs, neurological examination, etc
ECOG grade	Record the score
For HLA typing and testing	\
HLA antibody	\
Homicidal immunoglobulin-like receptors (KIR)	\
routine blood test	Red blood cell count (RBC), hemoglobin (HGB), platelet count (PLT), white blood cell count (WBC), absolute neutrophhil (Neut #), absolute lymphocytes (LYMPH #), etc
Blood biochemical	Albumin (ALB), alanine aminotransferase (ALT), aspartate aminotransferase (AST), alkaline phosphatase (ALP), glutamyl transpeptidase (GGT), total bilirubin (TBIL), direct bilirubin (DBIL), urea (Urea) or urea nitrogen (BUN), creatinine (CREA), creatine kinase (CK), lactate dehydrogenase (LDH), triglyceride (TG), cholesterol (CHOL), HDL cholesterol (HDL), low density lipoprotein cholesterol (LDL), Blood glucose (GLU), electrolytes (potassium, sodium, chlorine, calcium, magnesium, phosphorus), etc

List	Requirements and observation indicators
routine urine test	Urinary glucose (UGLU), ketone body (KET), urinary bilirubin (BIL), urinary biliary (UBG), urinary protein (PRO), pH (PH), ossitic blood (BLD), red blood cells (RBC), white blood cells (WBC), etc
Stool routine + occult blood	Appearance: color and characteristics; microscopic examination: white blood cells (WBC), red blood cells (RBC), pus cells, phagocytes; occult blood (OB), etc
A full set of coagulation	Prothrombin time (PT), International normalized ratio (INR), activated partial thromboplastin time (APTT), thrombin time (TT), fibrinogen (FIB)
Myocardial enzyme spectrum	Creatine kinase isozyme (CK-MB), myoglobin, troponin
BNP	Type B natriuretic peptide
Pregnancy examination	β Human chorionic gonadotropin (β -hCG)
Infectious disease detection	Two and a half (five) of hepatitis B, hepatitis B virus DNA (HBV-DNA), HCV antibody (HCV), hepatitis C virus (HCV), HCV virus RNA (HCV-RNA), Treponema pallidum antibody (TP-Ab), rapid plasma Reactor experiment, human immunodeficiency virus antibody (HIV (1 + 2) Ab)
cell factor	IL-2、IL-2R 、IL-4、IL-6、IL-8、IL-10、IL-15、TNF-α 、IFN-γ
ferritin	\
CRP	\
NK cell phenotype and function	Panel Index included CD45, CD33, CD14, CD3, CD56, and donor-specific HLA-antigen
Cerebrospinal fluid examination (test if necessary)	\
Blood smear examination	\
Bone marrow aspiration and / or bone marrow biopsy	Smear microscopy (bone marrow smear cytology), immunological examination (acute leukemia immunotyping), PK / PD testing of bone marrow samples, (genetic and chromosomal testing if necessary)
Bone marrow smear for cytology	\
Immunophenotypic analysis	\
Chromosome karyotyping (test if necessary)	\
FISH (test, if necessary)	\
AML-associated	MLL / ELL, MLL / AF17, MLL / AF6, MLL / AF9, MLL / AF10, AML

List	Requirements and observation indicators
fusion gene (tested if necessary)	1 / ETO, dupMLL, NPM / RAR α, PLZF / RAR α, PML / RAR α (bcr 1, bcr 2, bcr 3), DEK / CAN, NPM / MLF1, CBF β / MYH 11, TLS / ERG, EVI 1, HOX 11, BCR / ABL 1 (P190), BCR / ABL 1 (p210), etc
Gene mutations in AML (tested if necessary)	NPM 1, FLT 3, CEBPA, IDH 1 / 2, DNMT3A, KIT, TP 53, RUNX 1, ASXL 1, etc
Peripheral blood leukemia blast cells	\
echocardiogram	Ejection fraction (LVEF)
electrocardiogram	\
Radiological evaluation (if applicable)	\
Health-related quality of life assessment	See Annex 6
Adverse events and concomitant medication use	Adverse events and concomitant medication use were recorded

Bone Marrow Examination: Includes bone marrow aspiration and/or biopsy performed according to standard nursing procedures. Bone marrow examination items include smear microscopy (bone marrow cytology), immunological testing (acute leukemia immunophenotyping), and PK/PD testing of bone marrow samples during the screening period and on Days 28, 60, 90, 180, 270, and 360. A 0.2 mL bone marrow sample is required for smear microscopy, and a 2-3 mL sample is required for immunophenotyping and PK/PD testing of bone marrow samples. Other bone marrow testing may be performed as needed for the study, such as chromosome analysis (FISH and conventional karyotyping) and gene analysis (fusion gene and/or mutation gene testing by sequencing). If testing is performed, approximately 4 mL and 2-3 mL of bone marrow samples will be needed, respectively.

Echocardiography and Electrocardiography: Screening echocardiography and electrocardiography results within 14 days before signing the informed consent form.

4.2 Study Intervention Protocol

Participants will receive a single intravenous infusion of anti-CD33 CAR-NK cells, with the infusion to be completed within 30 minutes. The planned dose of anti-CD33 CAR-NK cells is 1.0×10^9 cells, and 15-20 participants are expected to be enrolled.

4.2.1 Pre-Treatment

Cell Production and Quality Control Period (Day -18 to Day -1)

The production and quality control of anti-CD33 CAR-NK cells are expected to take approximately three weeks. During this period, adverse events and concomitant medications will be recorded.

4.2.2 Chemotherapy Period (Day -5 to Day -3)

Height and weight will be measured before chemotherapy.

A blood pregnancy test will be performed before the first chemotherapy session (for women of childbearing age only).

Participants will receive lymphodepleting chemotherapy: fludarabine 30 mg/m^2 (Day -5 to Day -3) and cyclophosphamide 300-500 mg/m^2 (Day -5 to Day -3).Adverse events and concomitant medications will be recorded.

4.3 Data Collection

4 4.3.1 Chemotherapy Recovery Period (Day -2 to Day -1)

On Day -1, the following observations and assessments must be completed for the participant:

1) Vital signs
2) General physical examination
3) ECOG performance status
4) Complete blood count (CBC)
5) Blood biochemistry
6) Urinalysis
7) Stool routine + occult blood test (This will be determined based on the participant's specific condition, but the test must be conducted at least once within 5 to 7 days before CD33 CAR-NK cell infusion)
8) Coagulation profile

9) Cardiac enzyme profile

10) Cytokine profile

11) Ferritin

12) C-reactive protein (CRP)

13) Peripheral blood smear

14) Bone marrow aspiration and/or biopsy

15) Bone marrow cytology

16) Peripheral blood leukemic blasts

17) Electrocardiogram (ECG)

18) Recording of adverse events and concomitant medications

A total of six tocilizumab vials should be prepared one day before the infusion of CD33 CAR-NK cells. Based on the results of the above assessments, the investigator will determine whether the participant is eligible to receive CD33 CAR-NK cell therapy or if treatment should be delayed. If the participant shows disease progression after apheresis but before the infusion of CD33 CAR-NK cells, the investigator will determine whether to administer bridging chemotherapy (systemic corticosteroids at doses >10 mg/day of prednisone or equivalent are prohibited). If the participant receives chemotherapy, a 7-day washout period is required before the investigator reassesses the participant's eligibility for lymphodepleting chemotherapy and CD33 CAR-NK cell infusion.

Follow-up Schedule:

4.4 Day 0

1) Measure height and weight before cell infusion
2) Vital signs: Begin ECG monitoring 1 hour before CD33 CAR-NK cell infusion. Monitor every 30 minutes ± 5 minutes from 1 hour before the infusion until 2 hours after the infusion ends. Afterward, it is recommended to monitor every hour ± 5 minutes for 2 hours (Day 0).
3) General physical examination
4) Pre-medication: Before CD33 CAR-NK cell infusion, the investigator may choose

to administer acetaminophen and diphenhydramine or other H1-antihistamines.

5) Administration of CD33 CAR-NK cell therapy
6) Blood sampling for cell pharmacokinetics: Blood samples should be taken 30 minutes before the CD33 CAR-NK cell infusion and 1 hour after the infusion ends.
7) Blood sampling for immunogenicity: Blood should be drawn 30 minutes before CD33 CAR-NK cell infusion.
8) Record adverse events and concomitant medications
9) Dose-limiting toxicity (DLT) assessment (only during dose-escalation phase)

4.5 First Month Post-Infusion

4.5.1 Weekly Visits During the First Week (Days 1, 3, 5, 7)

1) Vital signs: Monitor every 2 hours ± 30 minutes during the first 24 hours (Day 1) post-infusion and every 3 hours ± 30 minutes during the first 48 hours (Day 2). Monitoring duration may be extended based on the participant's condition.
2) General physical examination
3) Complete blood count (CBC)
4) Blood biochemistry
5) Urinalysis
6) Stool routine + occult blood test (determined based on the participant's condition, but must be performed at least once within 7 days post-CD33 CAR-NK cell infusion)
7) Coagulation profile
8) Cardiac enzyme profile
9) Cytokine profile
10) Ferritin
11) C-reactive protein (CRP)
12) Electrocardiogram (ECG)
13) Blood sampling for cell pharmacokinetics on Days 3 and 7 post-infusion
14) Blood sampling for immunogenicity on Day 7 post-infusion
15) Record adverse events and concomitant medications
16) Dose-limiting toxicity (DLT) assessment (only during dose-escalation phase)

In addition to the above, ECOG performance status should be assessed after CD33 CAR-NK cell infusion.

4.5.2 Visits During Weeks 2-4 Post-Infusion (Day 14 ± 3, Day 21 ± 3, Day 28 ± 3)

1) Vital signs
2) General physical examination
3) ECOG performance status
4) Complete blood count (CBC)
5) Blood biochemistry
6) Urinalysis
7) Stool routine + occult blood test (determined based on the participant's condition)
8) Coagulation profile
9) Cardiac enzyme profile
10) Cytokine profile
11) Ferritin
12) C-reactive protein (CRP)
13) Peripheral blood smear
14) Peripheral blood leukemic blasts
15) Minimal residual disease (MRD) testing (to be conducted when CR is suspected and approximately 6 and 12 months from the start of the study)
16) Electrocardiogram (ECG)
17) Blood sampling for cell pharmacokinetics on Days 14, 21, and 28 post-infusion
18) Blood sampling for immunogenicity on Days 14 and 28 post-infusion
19) Record adverse events and concomitant medications
20) Dose-limiting toxicity (DLT) assessment (only during dose-escalation phase)

Additionally, on Day 28 ± 3 days post-CD33 CAR-NK cell infusion, the following assessments should be performed: HLA antibody testing, bone marrow aspiration and/or biopsy, bone marrow cytology, efficacy evaluation, replication-competent lentivirus (RCL) testing, echocardiography, and health-related quality of life (HRQoL) assessment.

4.5.3 Months 2-12 Post-Infusion

Participants will undergo safety and efficacy assessments according to the study protocol: from Month 2 to Month 6 post-infusion, assessments will be conducted every 30 days (i.e., on Days 60, 90, 120, 150, and 180), with a time window of ± 3 days. From Month 7 to Month 12 post-infusion, assessments will be conducted every 90 days (i.e., on Days 270 and 360), with a time window of ± 7 days.

The following evaluations will be conducted during treatment and follow-up visits:

1) Vital signs
2) General physical examination
3) ECOG performance status
4) HLA antibody testing (if efficacy is not deemed CR, the specific time will be determined by the investigator)
5) Complete blood count (CBC)
6) Blood biochemistry
7) Urinalysis
8) Stool routine + occult blood test (determined based on the participant's condition)
9) Cytokine profile
10) Ferritin
11) Peripheral blood smear
12) Bone marrow aspiration and/or biopsy
13) Bone marrow cytology
14) Peripheral blood leukemic blasts
15) Minimal residual disease (MRD) testing (to be conducted when CR is suspected and approximately 6 and 12 months from the start of the study)
16) Efficacy evaluation
17) Health-related quality of life (HRQoL) assessment
18) Replication-competent lentivirus (RCL) testing
19) Blood sampling for cell pharmacokinetics on Days 60, 90, 120, 150, 180, 270, and 360 post-infusion
20) Blood sampling for immunogenicity on Days 60, 90, 120, 150, 180, 270, and 360 post-infusion

21) Record adverse events and concomitant medications

4.5.4 Exit Visit (Within 7 Days of Withdrawal)

For participants who withdraw early from the study, an exit visit should be scheduled within 7 days of the decision to withdraw. The timing of specific assessments will be determined by the investigator based on actual circumstances. If laboratory tests, ECG, and echocardiography were completed within 7 days before withdrawal, these tests do not need to be repeated during this phase.

1) Vital signs
2) General physical examination
3) ECOG performance status
4) Complete blood count (CBC)
5) Blood biochemistry
6) Urinalysis
7) Stool routine + occult blood test (determined based on the participant's condition)
8) Ferritin
9) Bone marrow aspiration and/or biopsy
10) Bone marrow cytology
11) Peripheral blood leukemic blasts
12) Minimal residual disease (MRD) testing (to be conducted when CR is suspected and approximately 6 and 12 months from the start of the study)
13) Efficacy evaluation
14) Record adverse events and concomitant medications

4.5.5 Safety Follow-up

If the participant completes the Day 360 visit or withdraws from the study due to disease progression or intolerable toxicity, safety follow-up will be conducted. The overall safety observation period is one year after CAR-NK cell infusion, with follow-up every three months (either by an in-clinic visit or phone call, depending on the participant's condition). Follow-up will continue until one year after CAR-NK cell infusion, the participant's death, or the initiation of another anti-tumor therapy. Safety data related to cell therapy will be collected. If the participant cannot visit

4.5.6 Unscheduled Visits

During the study, for the safety of the subjects, if adverse events (AEs) or abnormal laboratory results occur, the investigator may increase the frequency of follow-up visits as needed, constituting unscheduled visits. The investigator must accurately record each unscheduled visit in the subject's original documentation and in the unscheduled visit section of the electronic Case Report Form (eCRF).

5. Study Endpoints

Based on the 2003 International Working Group (IWG) response criteria (see Appendix 9), the therapeutic efficacy for the subjects will be evaluated.

Primary Efficacy Endpoints: Safety, dose-limiting toxicity (DLT), maximum tolerated dose (MTD), and recommended phase 1 dose (RP1D).

Secondary Efficacy Endpoints: Overall response rate (ORR), complete response rate (CRR), partial response rate (PRR), relapse-free survival (RFS), duration of response (DOR), 12-month progression-free survival (PFS) rate, 12-month overall survival (OS) rate, median PFS, median OS, and health-related quality of life.

5.1 Evaluation of Other Study Objectives

5.1.1 Cellular Pharmacokinetics Evaluation

Peripheral blood CAR transgene copy numbers and/or CAR-NK cell counts will be processed using WinNonlin® software (version 8.1 or later), and key pharmacokinetic parameters, including C_{max} (observed value), T_{max} (observed value), and AUC, will be estimated using a non-compartmental model. Descriptive statistical analysis of the central tendency and dispersion of pharmacokinetic parameters will be conducted using SAS software. The following pharmacokinetic parameters will be measured or calculated: T_{max}, C_{max}, AUC_{0-t}, $AUC_{0-\infty}$, and $t_{1/2}$.

5.2 Safety Evaluation

Safety assessments will be conducted during the screening period, throughout the study, and after the study concludes. Subjects who withdraw early should undergo a safety assessment prior to withdrawal.

Safety Evaluation Criteria: Adverse events (AEs), cytokine release syndrome (CRS),

vital signs, general physical examination, ECOG score, laboratory tests, electrocardiogram (ECG), and echocardiography.

1) Adverse Events: Definitions, assessments, and follow-up requirements for adverse events are detailed in Section 7.4.
2) Cytokine Release Syndrome: Including cytokines, ferritin, and CRP.
3) Vital Signs: As per the study schedule and procedures outlined in Section 5.6, including respiratory rate, pulse, blood pressure, oxygen saturation, and body temperature.
4) General Physical Examination: A complete physical examination will be conducted as per the study schedule and procedures outlined in Section 5.6, including assessment of the skin, mucous membranes, lymph nodes, head, neck, chest, abdomen, spine/limbs, and neurological system.
5) ECOG Score.
6) Laboratory Tests: As per the study schedule and procedures outlined in Section 5.6, including complete blood count, urinalysis, stool routine with occult blood, blood biochemistry, coagulation profile, and cardiac enzymes.
7) Electrocardiogram (ECG): A resting ECG will be performed as per the study schedule and procedures outlined in Section 5.6. For the safety of the subjects, additional ECGs may be performed as needed during the study.
8) Echocardiography: As per the study schedule and procedures outlined in Section 5.6.

6. Data Management and Statistical Analysis

This trial is a prospective clinical study evaluating the safety and efficacy of anti-CD33 CAR-NK cell therapy for relapsed/refractory acute myeloid leukemia (AML). Six months after the last subject is enrolled, statistical analyses will be performed on primary and secondary endpoints, as well as safety data. All statistical analyses will be conducted using statistical software (SAS). The efficacy results will primarily involve statistical inference, while safety results will be primarily descriptive.

ALTERNATIVE TREATMENT OPTIONS

All patients are end-stage AML patients, with a conventional treatment 5-year survival

rate of only 10%.

OBSERVATION, RECORDING AND HANDLING THE ADVERSE EVENTS

Definition of Adverse Events

An adverse event (AE) refers to any unfavorable medical occurrence in a subject following the administration of the investigational product, which may present as symptoms, signs, diseases, or abnormal laboratory findings. An AE may not necessarily have a causal relationship with the investigational product.

Definition of Serious Adverse Events (SAEs)

A serious adverse event (SAE) is defined as any AE that meets one or more of the following criteria:

1) Results in death;
2) Is life-threatening*;
3) Requires hospitalization or prolongation of existing hospitalization;
4) Results in persistent or significant disability/incapacity;
5) Causes a congenital anomaly/birth defect;
6) Other important medical events that, based on medical judgment, may not be immediately life-threatening, fatal, or result in hospitalization but may jeopardize the subject or require intervention to prevent one of the above outcomes.

*Note: The definition of "life-threatening" refers to an AE in which the subject is at immediate risk of death at the time of the event, not one that may potentially result in death if left untreated.

Recording and Describing Adverse Events/Serious Adverse Events

All observed or subject-reported AEs during the study must be accurately documented by the investigator in the original medical records and entered into the eCRF under the "Adverse Events" section. The recording and description of AEs should adhere to the following principles:

1) Completeness: The original medical record should include, but is not limited to, the basic information of the trial and subject, details of anti-CD33 CAR-NK cell use, the occurrence of the AE, treatment measures taken, actions taken regarding anti-CD33 CAR-NK cells, the outcome of the AE, causality assessment and

rationale, and concomitant medications.

2) Readability: Avoid abbreviations for medical terminology to reduce ambiguity.

The information recorded should include, but is not limited to:

- AE name;
- Start date;
- Severity;
- Actions taken;
- Actions taken with anti-CD33 CAR-NK cells;
- Causality with anti-CD33 CAR-NK cells;
- Whether it is a serious adverse event;
- Outcome of the AE;
- End date;
- Whether the subject withdrew from the clinical study due to the AE.

Determining the Name of Adverse Events/Serious Adverse Events

The name of the AE should be a medical term, with a preference for using a medical diagnosis. If multiple symptoms, signs, and laboratory abnormalities can be grouped as part of a disease or injury, they should be considered a single AE. If a diagnosis cannot be made, symptoms/signs should be used, and when a diagnosis becomes clear later, the records should be updated to reflect the diagnosis instead of the symptoms/signs.

When determining the name of the AE, ensure that each name consists of a single event. One diagnosis or symptom/sign equals one AE.

Terms such as hospitalization, surgery, or death are not AEs; the cause of these events should be recorded as the AE. If the cause is unclear, known information such as hospitalization or death can be used as the AE name, which will later be updated with more detailed information during follow-up.

Grading the Severity of Adverse Events/Serious Adverse Events

Investigators should refer to the NCI CTCAE (version 5.0) to evaluate the severity of AEs. If the guideline does not provide specific severity grading for a certain AE,

investigators may assess the severity based on the general definitions of grades 1-5 and their clinical judgment. General AE grading includes:

Grade 1: Mild; asymptomatic or mild symptoms; only clinical or diagnostic observations; no intervention required.

Grade 2: Moderate; minimal, local, or noninvasive intervention required; limiting instrumental activities of daily living (ADLs)*.

Grade 3: Severe or medically significant but not immediately life-threatening; hospitalization or prolongation of hospitalization indicated; disabling; limiting self-care ADLs**.

Grade 4: Life-threatening; urgent intervention required.

Grade 5: Death related to AE.

*Instrumental ADLs include preparing meals, shopping for clothing, using the telephone, and managing finances.

**Self-care ADLs include bathing, dressing, feeding oneself, toileting, taking medications, and not being bedridden.

Handling of Serious Adverse Events

3.4.1 Reporting Serious Adverse Events

Investigators should not delay reporting to ethics committees or regulatory authorities while awaiting additional data to complete the records. Initial reports should contain sufficient information to clarify the following:

1) Subject's trial ID number;
2) Subject's name in pinyin initials;
3) Date and time of first administration of cell therapy;
4) Date and time of the event;
5) Brief description of the event and countermeasures taken;
6) Investigator’s opinion on the relationship between the event and cell therapy.

Following the initial fax report, a more detailed report on the serious adverse event (SAE) should be completed using the SAE section of the case report form (CRF). This report should clarify any ambiguous aspects of the event and include, whenever possible, relevant hospital records and autopsy reports.

The investigator must report SAEs to regulatory authorities (if applicable) and the Institutional Review Board (IRB) in accordance with local laws and regulations.

Follow-Up of Serious Adverse Events

All SAEs must be followed up until they are deemed resolved, stabilized, the subject is lost to follow-up, or the event can be otherwise explained. The frequency of follow-up assessments should be determined by the investigator based on the specifics of the SAE.

Assessment of Expectedness of Serious Adverse Events

The assessment of expectedness should be based on serious adverse reactions previously observed in the clinical study. In subsequent phases of the clinical study, the determination of whether an SAE is expected will rely on the safety reference information provided in the Investigator's Brochure, specifically in the section listing expected serious adverse reactions (refer to the most recent version of the Investigator's Brochure for safety reference information).

Disease Progression

Clear signs or symptoms of tumor progression should not be recorded as adverse events (if supported by diagnostic evidence) unless they are more severe than expected, or the investigator believes that the tumor progression is related to the investigational drug or study procedure. In cases of newly diagnosed primary malignancies, these events should be reported as SAEs.

All deaths occurring during the trial, including those due to disease progression, must be reported as SAEs.

QUALITY CONTROL

Before the initiation of the study, all investigators must undergo protocol training. During the study, strict adherence to the protocol and relevant Standard Operating Procedures (SOPs) is required. Investigators must carefully review all records, verify

data, and ensure the accuracy, integrity, and completeness of the study data, while safeguarding the safety and rights of the participants. The following requirements must be met:

1) Original documents must comply with China's Good Clinical Practice (GCP) guidelines.
2) Laboratory test results must be truthful, accurate, reliable, and complete.
3) All observations and findings should be verified to ensure data reliability.
4) All staff involved in the study must strictly follow the protocol and procedures without altering records arbitrarily.
5) A designated statistician will be responsible for comprehensive statistical analysis of the data.
6) After the study is completed, the electronic Case Report Forms (eCRFs) will be saved on a disk and stored at the research center.

DATA SAFETY MONITORING

The clinical study will implement a data safety monitoring plan based on the level of risk involved. All adverse events will be recorded in detail, appropriately handled, and followed up until resolution or stabilization. Serious adverse events (SAEs) and unexpected events will be reported promptly to the Ethics Committee, regulatory authorities, the sponsor, and the drug supervision department as required. The principal investigator will regularly review cumulative adverse events, and if necessary, convene an investigator meeting to evaluate the risk-benefit ratio of the study. In double-blind trials, emergency unblinding may be performed if necessary to ensure the safety and rights of participants.

ETHICAL PRINCIPLES AND REQUIRMENTS

The clinical study will comply with the Declaration of Helsinki and the "Ethical Review Measures for Life Sciences and Medical Research Involving Humans" of the People's Republic of China. Specific requirements include informed consent, privacy protection, free participation and compensation, risk control, protection of vulnerable participants, and compensation for research-related injuries. The clinical study will only commence after approval of the study protocol by the Ethics Committee. Before enrolling any

participant, the investigator is responsible for fully explaining the purpose, procedures, and potential risks of the study to the participant and/or their legal representative. A written informed consent form must be signed, ensuring that participants are fully informed and voluntarily joining the study. Participants have the right to refuse participation or withdraw from the study at any time without facing discrimination or retaliation, and their medical treatment and rights will remain unaffected. The investigator is responsible for protecting the personal privacy and data confidentiality of participants. Study data will be stored at the Second Affiliated Hospital of Army Medical University. Due to legal/regulatory reasons, the following exceptions to privacy protection exist: the investigator, regulatory authorities, and the Ethics Committee may access the data.

The research team hereby commits to making every effort within the bounds of the law to protect the personal privacy and information of participants. Security measures for safeguarding the privacy and confidentiality of participant information include de-identification of participant data in reports, restricting access to personal data, and data anonymization. The results of this study will be reported without disclosing any participant's personal identity.

RESEARCH PLAN

November 2021 to November 2022 (Expected)
